# Spastic Paraplegia Mutation N256S in the Neuronal Microtubule Motor KIF5A Disrupts Axonal Transport in a *Drosophila* HSP Model

**DOI:** 10.1371/journal.pgen.1003066

**Published:** 2012-11-29

**Authors:** Petra Füger, Vrinda Sreekumar, Rebecca Schüle, Jeannine V. Kern, Doychin T. Stanchev, Carola D. Schneider, Kathrin N. Karle, Katharina J. Daub, Vera K. Siegert, Matthias Flötenmeyer, Heinz Schwarz, Ludger Schöls, Tobias M. Rasse

**Affiliations:** 1 Junior Research Group Synaptic Plasticity, Hertie-Institute for Clinical Brain Research, University of Tübingen, Tübingen, Germany; 2 Graduate School of Cellular and Molecular Neuroscience, University of Tübingen, Tübingen, Germany; 3 German Center for Neurodegenerative Diseases (DZNE), Tübingen, Germany; 4 Hertie-Institute for Clinical Brain Research and Center for Neurology, Department of Neurodegenerative Disease, University of Tübingen, Tübingen, Germany; 5 Max Planck Institute for Developmental Biology, Tübingen, Germany; The University of North Carolina at Chapel Hill, United States of America

## Abstract

Hereditary spastic paraplegias (HSPs) comprise a group of genetically heterogeneous neurodegenerative disorders characterized by spastic weakness of the lower extremities. We have generated a *Drosophila* model for HSP type 10 (SPG10), caused by mutations in KIF5A. KIF5A encodes the heavy chain of kinesin-1, a neuronal microtubule motor. Our results imply that SPG10 is not caused by haploinsufficiency but by the loss of endogenous kinesin-1 function due to a selective dominant-negative action of mutant KIF5A on kinesin-1 complexes. We have not found any evidence for an additional, more generalized toxicity of mutant Kinesin heavy chain (Khc) or the affected kinesin-1 complexes. Ectopic expression of *Drosophila* Khc carrying a human SPG10-associated mutation (N256S) is sufficient to disturb axonal transport and to induce motoneuron disease in *Drosophila*. Neurofilaments, which have been recently implicated in SPG10 disease manifestation, are absent in arthropods. Impairments in the transport of kinesin-1 cargos different from neurofilaments are thus sufficient to cause HSP–like pathological changes such as axonal swellings, altered structure and function of synapses, behavioral deficits, and increased mortality.

## Author Summary

Hereditary spastic paraplegias (HSPs) comprise a group of inherited neurological diseases. The main feature of HSP is progressive stiffness of the lower limbs due to a dysfunction of nerve cells. We study HSP type 10, which is caused by mutations in the neuronal motor protein KIF5A. HSP type 10 is inherited in an autosomal-dominant manner, which means that patients have a normal and a mutated copy of the *KIF5A* gene. KIF5A plays an important role in neuronal function: it transports cargos to the synapse that can be up to 1 m from the cell body. We use the fruit fly as a model to investigate the role of mutations in KIF5A. Our fly model replicates a central feature of HSP: muscles that are activated by nerve cells that have long cellular processes are more severely impaired. We now address why one mutated copy of KIF5A is sufficient to cause HSP. To date, it has been thought that patients might have HSP because they have insufficient functional KIF5A or because mutated KIF5A disturbs the function of normal KIF5A. We provide evidence for the latter possibility.

## Introduction

Hereditary spastic paraplegia (HSP) is a group of genetically heterogeneous neurodegenerative disorders characterized by distal axonopathy that affects the longest axons in the corticospinal tract [1], [2]. To date, 48 HSP loci have been described. The three most common causes of HSP - accounting for more than 50% of all cases - are mutations in SPG3A (*Atlastin*), SPG4 (*Spastin*) and SPG31 (*Reep1*). Both *Atlastin* and *Spastin* mutations as well as mutations in 6 other identified SPG genes: (*KIF5A*, *Nipa*, *Spatacsin*, *Spastizin*, *Spartin* and *Maspardin*) have been implicated in disturbances of the intracellular transport. This suggests that perturbations in long-range, tubulin based transport might be a common pathological mechanism underlying different forms of HSP (for review see [3]).

SPG10 is inherited in an autosomal-dominant manner, with age of onset varying from childhood to the fourth decade of life [4]. The SPG10 gene KIF5A encodes the heavy chain of the neuronal microtubule motor kinesin-1 [5], [6]. The kinesin-1 family is the major anterograde motor complex.

To date, 21 different SPG10 mutations have been described [5], [6], 19 of which localize to the motor domain of KIF5A. Neither genomic deletions nor “truncating” mutations were identified as causes of autosomal-dominant SPG10, suggesting that SPG10 may be caused by a dominant-negative effect rather than by haploinsufficiency.

We generated a *Drosophila model* for SPG10 to validate the proposed dominant-negative interaction between mutant and wild-type Khc in the context of a living organism. Our results imply that SPG10 is not caused by haploinsufficiency but by the loss of endogenous kinesin-1 function. Thereby, Khc^N262S^ acts as an antimorph, not as a neomorph.

## Results

### Establishment of Khc^N262S^-expressing flies

In a previous *in vitro* study, four point mutations in the *KIF5A* gene that cause HSP in humans were analyzed [7]. One of these mutations, N256S, caused a reduction of motor velocity and displayed a dominant-negative effect on the function of wild-type KIF5A at physiologically relevant ratios of mutated and wild-type kinesin, but did not influence its microtubule binding affinity [7]. The N256S mutation was selected to generate the first *in vivo Drosophila* model for SPG10. Using this model we aimed at demonstrating that the mutated protein is stable in the context of an intact organism. Consistent with *in vitro* results [7] human genetic studies suggest [5], [6] that SPG10 is not caused by haploinsufficiency but by the dominant-negative interaction of mutated and wild-type kinesin. Thus, we wanted to prove that the dominant-negative action of mutated kinesin persists *in vivo* at physiologically relevant ratios, and is not abolished by cellular quality control mechanism, which might either prevent the hetero-dimerization of mutant and wild-type kinesin or which might selectively destabilize these dimers.

**Figure 1:**
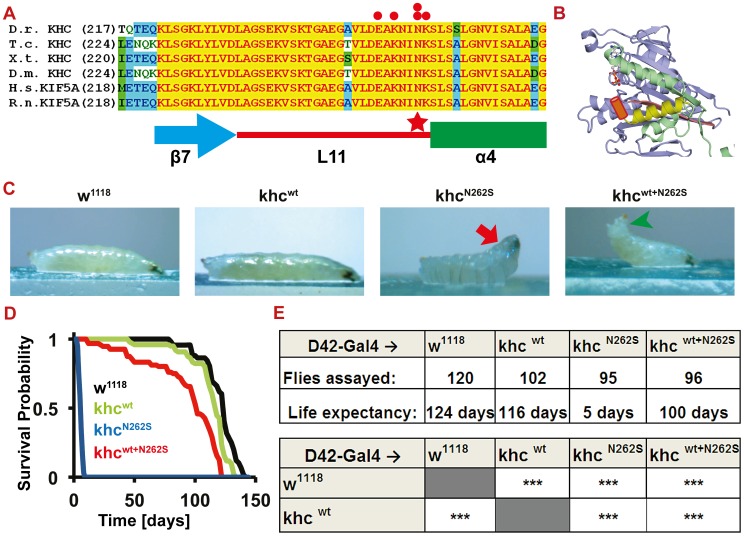
Establishment of a *Drosophila* SPG 10 model. (A) Khc protein alignment across species (*Danio rerio* (Dr), *Tribolium castaneum* (Tc), *Xenopus tropicalis* (Xt), *Drosophila melanogaster* (Dm), *Homo sapiens* (Hs), *Rattus norvegicus* (Rn)) shows unique conservation of the amino acids spanning from the β-sheet 7 (β7) to the α-helix 4 (α4). Pathological mutations (red dots) are highly enriched in the latter half of loop 11 (L11) that links β7 to α4. The human mutation at position 256 (red star) was selected for further analysis. Accession numbers for the proteins used in the alignment are: Dr: GenBank_CAQ15489.1; Tc: EFA10675.1; Xt: NCBI_NP_001096215.1; Dm: GenBank_AAF58029.1, Hs: NCBI_NP_004975.2; Rn: EDM 16486.1 (B) 3D protein structure of rat Khc (3KIN) [8]. The microtubule binding site is shown in yellow and green. L11 (brown cylinder), which was not resolved in the crystal structure, was added between β7 (yellow) and α4 (pink). (C) Analysis of larval locomotion. Larvae overexpressing D42>Khc^N262S^ (25°C) are almost completely (excluding the head) paralyzed (red arrow), whereas D42>Khc^wt+N262S^ larvae are still crawling, but display a tail-flipping phenotype (green arrowhead). (See also Videos S1, S2, S3) (D) Kaplan-Meier survival curve recorded at 18°C (Genotypes: black: D42>w^1118^; Green: D42>Khc^wt^; Blue: D42>Khc^N262S^; Red: D42>Khc^wt+N262S^) (E) Summary of the data derived from the survival analysis shown in (D). Statistical significance of data was determined by a series of Mantel-Cox tests. *** p<0.001.

The *KIF5A^N256S^* mutation, which corresponds to the amino acid exchange N262S in *Drosophila kinesin heavy chain (khc)* (Figure 1A, red star), is located on loop 11. Loop 11 (Figure 1A, red line; Figure 1B, brown cylinder) connects the microtubule- (α-helix 4: Figure 1A, green box; Figure 1B, yellow helix) and the ATP-binding site (β-sheet 7: Figure 1A, blue arrow; Figure 1B, pink β-sheet 7) of Khc [8]. Five of the 21 described human mutations in *KIF5A* (Figure 1A, red dots) map to seven amino acids (EAKNINK) of loop 11 [9], highlighting the importance of this structure, which is essentially 100% conserved between nematodes, arthropods, and chordates (Figure 1A).

### Khc^N262S^ expression causes HSP–like pathological symptoms

We used two complementary approaches to address the putative dominant negative action of Khc^N262S^ in *Drosophila*. Both involve - unlike previous studies of loss of function alleles [10]–[16] - the ectopic expression of Khc in the wild-type background. Therefore, pathological alterations should only occur if Khc^N262^-Khc^wt^ heterodimers are dysfunctional or if dimers containing Khc^N262S^ are directly toxic. Ectopic expression of Khc^N262S^ was induced either alone or in combination with Khc^wt^. The phenotypic severity of an antimorphic mutation will be decreased by increasing wild-type gene dosage. If Khc^N262S^ acts as an antimorph, coexpression of Khc^wt^ should ameliorate all observed defects.

Strong expression of Khc^N262S^ in motoneurons (D42-Gal4) of wild-type larvae raised at 25°C or 29°C caused severe pathological symptoms, resulting in the death of larvae in the L2 or L3 stage, respectively. Initially, larvae display the characteristic tail-flipping phenotype originally described for *khc* null mutant larvae [13]. As the paralysis progresses, it ascends from posterior to anterior until larvae can only move their head (Figure 1C, red arrow; Video S1). Complete paralysis and death ensue. This “ascending” paralysis mirrors human pathological symptoms characterized predominantly by affliction of the lower limbs due to the particular vulnerability of the long descending spinal tracts. In *Drosophila*, axons innervating the posterior segments are considerably longer than those innervating anterior segments and seem to be - just as in humans - primarily affected.

In larvae coexpressing both Khc^N262S^ and Khc^wt^ (Khc^wt+N262S^), phenotypes are ameliorated, but still persist. This suggests that Khc^N262S^ acts as an antimorph, not as a neomorph. At the age at which Khc^N262S^-expressing larvae are only able to move their head, Khc^wt+N262S^ larvae can still locomote while displaying a tail-flipping phenotype (Figure 1C, green arrowhead; Video S2). This delayed paralysis of Khc^wt+N262S^ larvae is progressive but does not cause larval lethality (Figure 1C). Khc^wt^ overexpression alone does not cause any obvious defects at either 25°C (Figure 1C; Video S3) or 29°C.

When the flies were raised at 18°C, decreased expression levels of Khc^N262S^ or Khc^wt+N262S^ resulted in extremely short-lived flies or flies with a 25% reduction in life span (Figure 1D). Expression of Khc^wt^ led to only a minimal but significant reduction in life span (Figure 1D, 1E).

**Figure 2:**
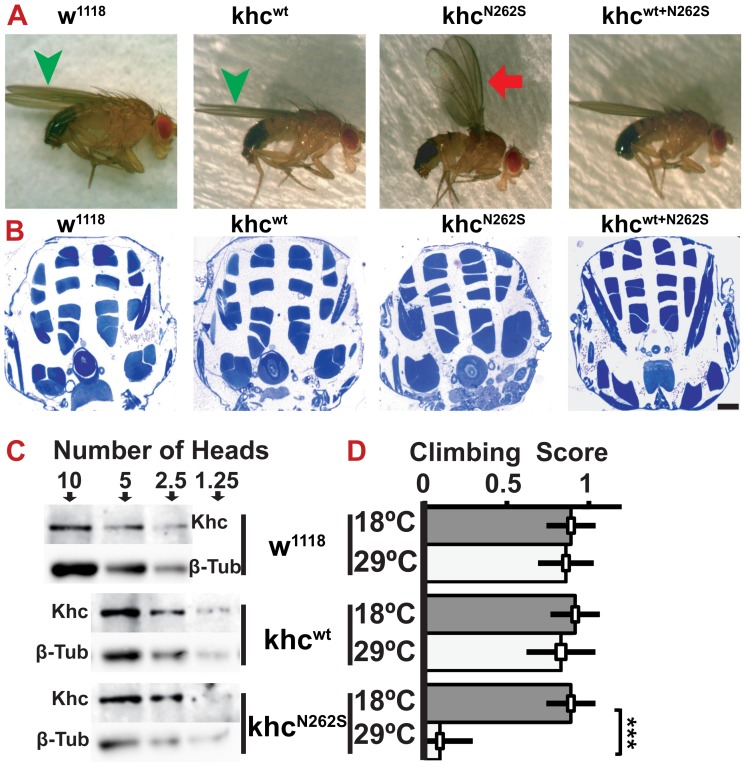
Characterization of adult *Drosophila* SPG 10 model flies. (A) Analysis of adult wing posture. While control flies have a normal wing posture (green arrowheads), Khc^N262S^ expressing flies frequently display abnormal wing postures such as holding their wings up (red arrow), suggesting either the degeneration or functional impairment of the indirect flight muscles. 2-day old male flies raised at 18°C are shown. All flies contained a copy of the motoneuron specific driver D42-Gal4. (B) Analysis of thorax muscle integrity provided no evidence for the degeneration of the indirect flight muscles. Scale bar: 100 µm. (C) Western blot of whole fly head extracts after adult-onset pan-neuronal (elav^C155^-Gal4/tub-gal80ts) expression of Khc^N262S^ and Khc^wt^ at 29°C for 13 days. Levels of endogenous Khc from 10 heads of control flies (w^1118^) are comparable to Khc levels in 2.5 heads from flies overexpressing Khc (Khc^N262S^ and Khc^wt^). The number of heads loaded per lane is indicated. β-Tubulin was used as loading control. (D) Climbing assay of adult flies 16 days after adult-onset pan-neuronal expression of Khc (white bars). Although Khc^wt^ expression caused no adverse effects on climbing scores compared with control (grey bars), Khc^N262S^ expression significantly lowered the climbing score. Statistical significance was tested using an unpaired, two-tailed student's t-test (*** p<0.001). The standard error of the mean (s.e.m) is shown as a box, standard deviation (s.d.) as a black line.

Furthermore, the behavior of flies expressing Khc^N262S^ at 18°C was strongly impaired. If forced to fly, animals could not sustain stable flight, but fell to the ground. At rest, they held their wings in an abnormal position (Figure 2A). Whereas control flies (Figure 2A, green arrowhead) held their wings parallel to their body axis, Khc^N262S^-expressing flies held their wings up (Figure 2A, red arrow). This behavior has been previously described in Parkinson disease-related *Drosophila pink-1* mutants, in which the wing posture defect was attributed to apoptotic degeneration of the indirect flight muscles [17]. However, we did not observe signs of muscle loss in Khc^N262S^-expressing flies when thoracic indirect flight muscles were analyzed (Figure 2B), suggesting that the abnormal wing posture is secondary to impaired motoneuron function and not a result of muscle degeneration.

In the above-described models, Khc^N262S^ is expressed throughout development. To test whether conditional expression of Khc^N262S^ (elav-Gal4, tub-Gal80^ts^) after completion of development is sufficient to cause neurodegeneration, we induced expression 0 to 24 hours after eclosion. Thirteen days of overexpression at 29°C led to a 3–4 fold excess of ectopic Khc compared with endogenous Khc (Figure 2C). Importantly, equal protein levels of Khc^wt^ and Khc^N262S^ were detected, showing that N262S does not affect protein stability (Figure 2C). Sixteen days after initiation of conditional overexpression, flies overexpressing Khc^N262S^ were essentially unable to climb a vertical plastic surface (Figure 2D), whereas flies overexpressing Khc^wt^ showed no obvious locomotion defects.

In summary, Khc^N262S^ acts as an antimorph, not as a neomorph. Either chronic or conditional expression of Khc^N262S^ is sufficient to cause HSP-like pathological changes in *Drosophila*.

### Expression of Khc^N262S^ disturbs axonal transport

**Figure 3:**
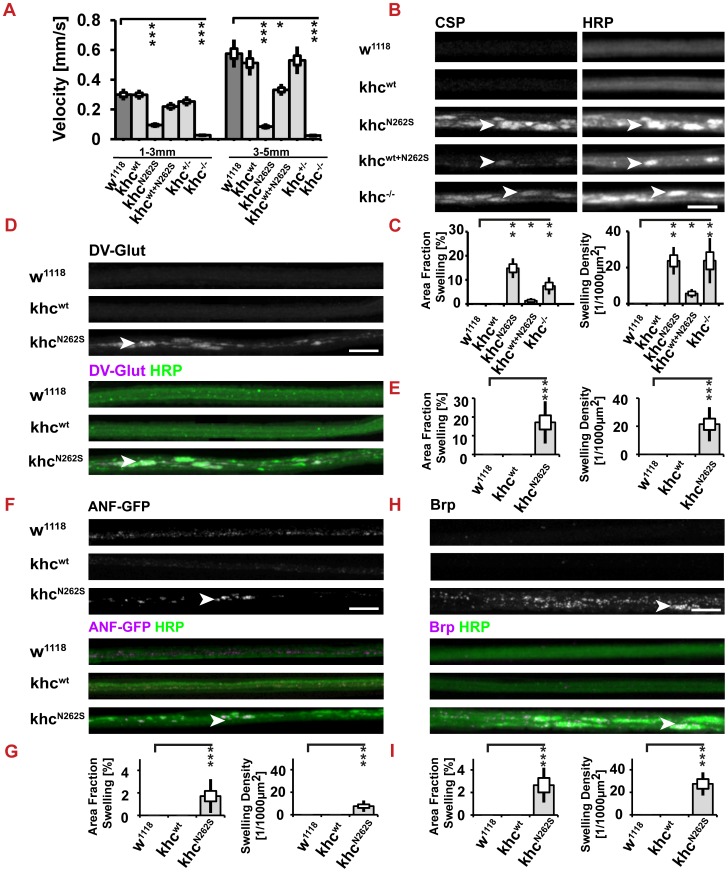
Analysis of larval locomotion and morphometric analysis of segmental nerves. Larvae used to assay locomotion were raised at 25°C. Larvae used for analysis of axonal cargo accumulations were raised at 29°C. All larvae (except khc^−/−^ and khc ^+/−^) carried one copy of the motoneuron-specific driver D42-Gal4. (A) Analysis of larval locomotion velocities. (B–I) Analysis of axonal cargo accumulation was performed by staining segmental nerves of third instar larva for the membrane marker anti-HRP and for various cargos. Axonal cargo accumulations (arrowheads in B,D,F,H) were defined as segments of the nerve characterized by a bright anti-HRP staining and the simultaneous accumulation of cargo. Both the area fraction of the nerve filled with cargo accumulations and the number of cargo accumulations per 1000 µm^2^ of the nerve were significantly increased in larvae expressing khc^N262S^, either alone, or in combination with Khc^wt^. (B,C) Cysteine-string protein (CSP) and (D,E) the vesicular glutamate transporter (DV-Glut) were selected as markers for synaptic vesicles. The kinesin-3 cargos (F,G) ANF-GFP and (H,I) Brp were used as markers for dense core vesicles and active zone precursor vesicles, respectively. Scale bars in B–I: 10 µm. For all quantifications, n = 7–10 axons per genotype were used. Statistical significance (A,C,E,G,I) was determined by using a Kruskal-Wallis H-test followed by a Dunn's test for comparisons between multiple groups. The standard error of the mean (s.e.m.) is shown as a box, the standard deviation (s.d.) as a black line. * p<0.05; ** p<0.01, *** p<0.001.

To further validate that Khc^N262S^ interferes with the function of wild-type Khc in a dominant-negative manner, we compared the effects of ectopic expression of Khc^N262S^ in a wild-type background to those observed in heterozygous and homozygous *khc* mutants. To this aim four to five day old larvae were selected. The expressions of Khc^N262S^ lead - likely due to a decreased overall fitness of these larvae - to delays in larval growth as exemplified by the decreased size of 120 h old larvae (D42>w^1118^: 3.3 mm, D42>khc^N262S^: 2.4 mm, p<0.001, Student's T-Test, two-tailed). D42>khc^N262S^ larvae showed also a trend to transit from the L2 to L3 stage being 15% smaller (data not shown). We thus decided to analyze the locomotion speed of larvae of the same size rather than of the same larval stage. This is reasonable as the length of a larva is the main biophysical parameter promoting or limiting the fast movement of a larva. The use of *Animaltracer* - a custom build algorithm - allowed us to determine both speed and length of larvae in a non-biased, automated manner. We next quantified the average speed of small (1–3 mm) and large (3–5 mm) larvae (Figure 3A). Locomotion of Khc^N262S^-overexpressing and *khc* deficient larvae was dramatically impaired compared with control and Khc^wt^-overexpressing larvae. Whereas locomotion of small Khc^wt+N262S^-expressing larvae was indistinguishable from that of controls, large larvae displayed a significant reduction in locomotion speed. No difference in the locomotion speed between wild-type larvae and larvae lacking one copy of *khc* were reported. These data support the hypothesis that SPG10 is caused by a dominant-negative action of mutated KIF5A that dramatically reduces endogenous kinesin-1 function. Consistent with the degenerative nature of HSP, locomotion defects caused by the ectopic expression of mutated Khc were generally more pronounced in larger larvae.

In the *khc* null mutant, the accumulation of the synaptic vesicle (SV) protein, cysteine string protein (CSP), has been reported [13]. We sought to address whether expression of Khc^N262S^ and of Khc^wt+N262S^ is sufficient to cause the accumulation of synaptic cargos in nerves. CSP has been reported to be a cargo of Khc [13], [18]. The expression of Khc^N262S^ and of Khc^wt+N262S^ led to the accumulation of cargos in axons (Figure 3B). Both the number and area fraction of nerves that were positive for cargo accumulations were highest in *khc* mutant larvae and in larvae overexpressing Khc^N262S^ (Figure 3C). Axonal cargo accumulations were absent in Khc^wt^-expressing larvae. Similar results were obtained by staining for another SV protein: the *Drosophila* vesicular glutamate transporter (DVGlut) (Figure 3D, 3E) [19], [20]. Accumulations of cargo coincided with an increased intensity of the neuronal membrane marker anti-HRP (arrowheads in Figure 3B, 3D, 3F, 3H).

Next, we scored for nonselective disturbances of axonal transport. Thereby we could show that both the transport of dense core vesicles (Figure 3F, 3G; visualized by ANF-GFP), [21] and the transport of the active zone protein Bruchpilot (Brp) [22], [23] is disturbed in Khc^N262S^ expressing larvae (Figure 3H, 3I). Both cargos are transported by the kinesin-3 family member *unc-104* [18], [24]; indicating that both the fast axonal transport of kinesin-1 and kinesin-3 cargos is disturbed by the expression of mutated Khc.

**Figure 4:**
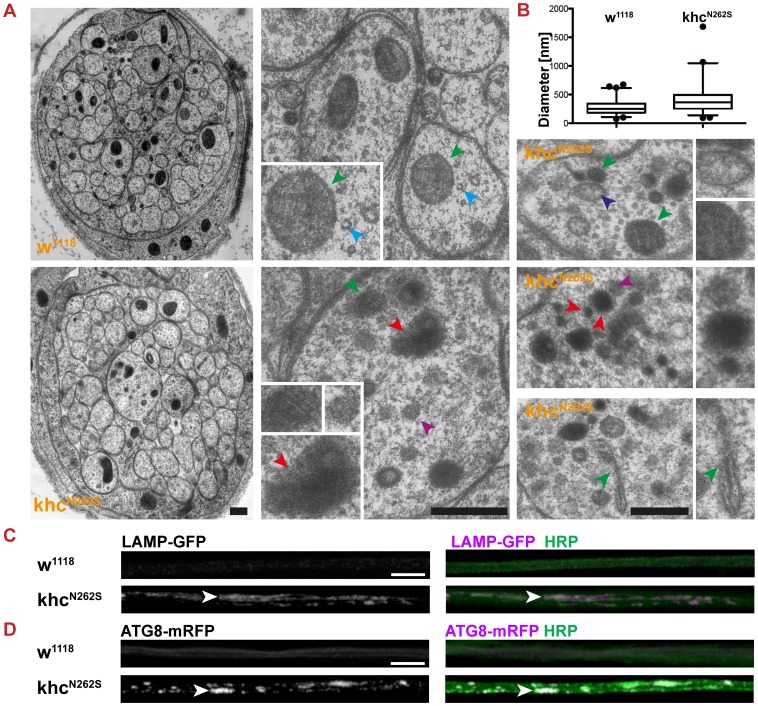
Characterization of axonal swellings. (A) Electron micrographs of segmental nerves of mid-L2 wild-type (D42>w^1118^) and mutant (D42>Khc^N262S^) larvae. Nerves of wild-type larvae contain mitochondria (green arrowhead) and microtubules (cyan arrowhead). Nerves of larvae expressing Khc^N262S^ are frequently swollen. These swollen axons are filled with mitochondria (green arrowheads), prelysosomal vacuoles (red arrowheads), autophagosomes (dark blue arrowheads), and multivesicular bodies (purple arrowheads). Scale bars: 100 nm. (B) Box-plot of axon diameters in mid-L2 wild-type (D42>w^1118^) and mutant (D42>Khc^N262S^) larvae as determined by electron microscopy. The box displays median, upper, and lower quartile. The whiskers represent the 1^st^ to 99^th^ percentile. (C,D) Confocal images of immunofluorescent staining showing segmental nerves of mid-third-instar *Drosophila* larvae. Larvae were stained for the membrane marker anti-HRP as well as for the lysosome marker LAMP-GFP (C) and the autophagosome marker ATG8-mRFP (D). Axonal swellings are positive for autophagosomes and lysosomal organelles. Scale bars in C and D: 10 µm.

We then aimed to validate that accumulations of cargo characterized by strong fluorescence for both anti-HRP and SV (Figure 3B, 3D, arrowhead) are axonal swellings. A swelling is defined by (1) the accumulation of cargo, (2) a local increase in anti-HRP staining intensity, and (3) a strong increase in axon diameter. Hurd and Saxton reported that at sites of axonal swellings, individual axons increase in diameter up to 10-fold within a micron [13]. We performed ultrastructural analysis to address whether axons were swollen upon Khc^N262S^ overexpression (Figure 4A). An example of a typical cross-section of nerves of control larvae and those expressing Khc^N262S^ is shown in the left panel of Figure 4A. The median axonal diameter was markedly increased in Khc^N262S^-expressing larvae (w^1118^: 0.25 µm, n = 320; Khc^N262S^: 0.36 µm, n = 301) (Figure 4B). Axons of control larvae contain microtubules (Figure 4A, cyan arrowhead), cargos of fast axonal transport such as mitochondria (Figure 4A, green arrowhead), and clear vesicles. As reported earlier for *khc* mutants [13], axonal swellings observed in Khc^N262S^-overexpressing larvae additionally contained large dark-staining organelles, including multivesicular bodies (Figure 4A, purple arrowhead), dark prelysosomal vacuoles (PLVs) (Figure 4A, red arrowhead), and autophagosomes (Figure 4A, dark blue arrowhead). To further validate the presence of lysosomal organelles in axonal swellings we expressed LAMP-GFP a marker for late endosomes and lysosomal compartments in motoneurons [25]. While no strong LAMP-GFP fluorescence was detected in control larvae, LAMP-GFP was strongly enriched in axonal swellings (Figure 4C, arrowhead) of Khc^N262S^-expressing larvae. The autosomal and autolysosomal marker ATG8-mrfp [26] also localized in axonal swellings (Figure 4D, arrowhead). The accumulation of PLVs can be triggered by impairments in the retrograde transport of small prelysosomal organelles, which then fuse and mature, giving rise to the PLVs observed in swellings [13]. Alternatively, stress-driven autophagy of the cytoplasm [27] might further contribute to the formation of PLVs [13]. The fact that the swellings are positive for ATG8 is consistent with the hypothesis that stress-driven autophagy of the cytoplasm might contribute to the formation of PLVs observed in electron microscopy [13].

### Expression of Khc^N262S^ disturbs cargo flux rather than velocity

We sought to use *in vivo* analysis of axonal transport to estimate disturbances in the delivery of cargo to synapses. The frequency at which organelles are delivered to synapses can be predicted by measuring cargo flux, i.e. the number of organelles that pass a defined cross-section of a nerve in a given time interval. Transport velocity allows estimation of how long it takes an organelle to reach its destination. A slow velocity might indirectly cause crowding in the axon. Upon a 50% reduction of cargo velocity, a 100% increase of cargo density is necessary to obtain the same flux.

Both genetic deletion of KIF5A [28] and ectopic expression of KIF5A^N256S^ [29] reduced anterograde and retrograde flux of neurofilaments in cultured mouse cortical neurons. Although no effects of overexpression of KIF5A^N256S^ on anterograde velocity were reported [29], deletion of KIF5A (KIF5A^−/−^) reduced both maximum and average velocities of neurofilament transport [28]. Neurofilaments were not depleted from distal axons upon overexpression of KIF5A^N256S^ [29]; neither axonal swellings nor increased apoptosis was reported [29]. In cultured KIF5A^−/−^ motoneurons [30], anterograde and retrograde transport velocities of mitochondria were reduced [30] compared with those of controls (KIF5A^+/+^). Effects on mitochondrial flux had not been investigated to date. We thus sought to determine the effects of deleting KIF5A on cargo flux. Anterograde (KIF5A^+/+^: 0.10±0.022 min^−1^; KIF5A^−/−^: 0.06±0.015 min^−1^, p = 0.045) but not retrograde flux of mitochondria (KIF5A^+/+^: 0.08±0.018 min^−1^; KIF5A^−/−^: 0.08±0.017 min^−1^, p = 0.93) was affected by loss of KIF5A. The number of stationary mitochondria detected within a 20 µm segment of the axon (KIF5A^+/+^: 3.11±0.75; KIF5A^−/−^: 2.83±0.429, p = 0.958) was not altered. Reductions in flux might be directly caused by reductions in transport velocity, or might be attributable to secondary defects. The 50% reduction of anterograde velocity that had been reported for KIF5A^−/−^ motoneurons [30] fully explains the observed 44% reduction in anterograde flux that we observed.

Although loss of *khc* [12] resulted in the reduction of retrograde flux rates in *Drosophila*, no impairment was observed in the KIF5A^−/−^ motoneuron culture model. Our measurements were performed in motoneurons isolated at day E12.5 and assessed at day 4 *in vitro*, an early developmental time point that corresponds to an early stage of pathological progression at which no retrograde depletion of cargo would occur. We suggest that secondary defects, e.g. impaired microtubule stability, the formation of axonal traffic jams, or the distal depletion of mitochondria, might contribute to reductions in retrograde flux observed in *khc* deficient larvae [12].

**Figure 5:**
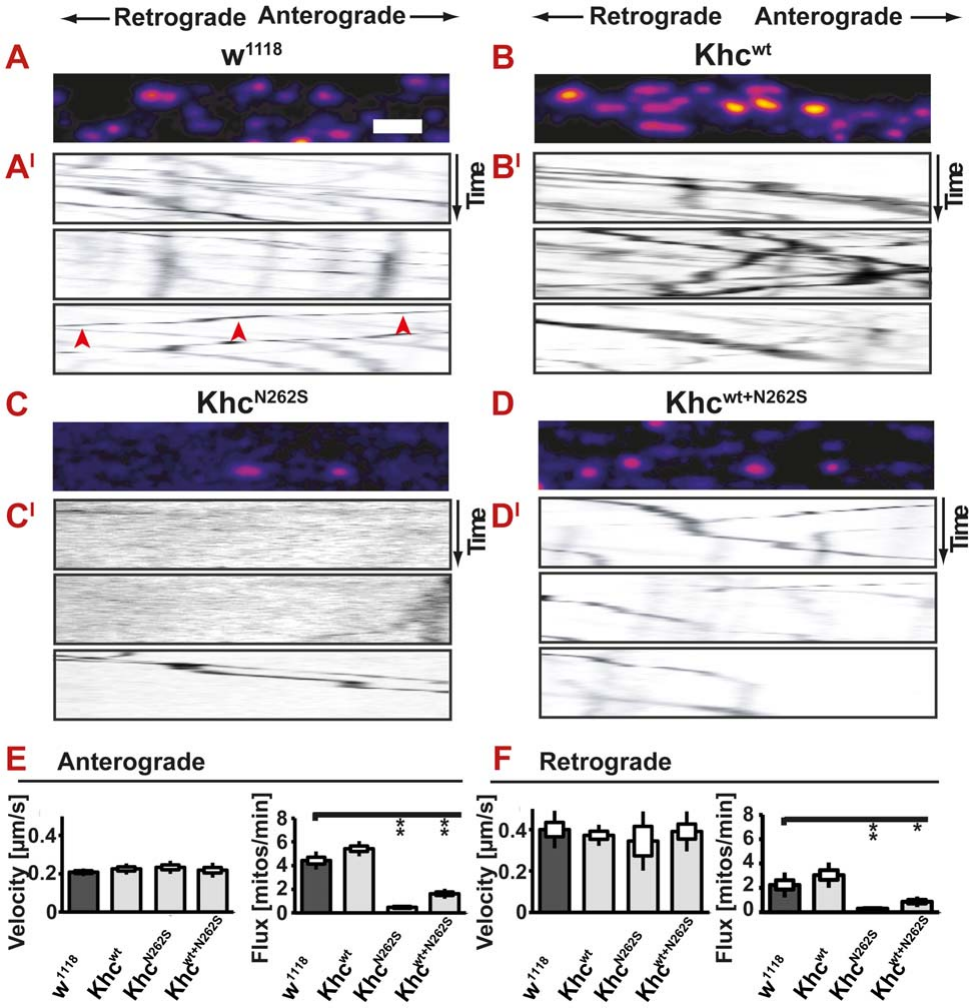
*In vivo* analysis of axonal transport. (A–D) Confocal analysis of mitochondrial transport in living larvae after bleaching a segment of the nerve. (A–D) Movement of mitochondria is recorded in anesthetized larvae. A representative frame of a confocal movie is shown. (A^I^–D^I^) Kymographs of mitochondrial transport in larvae. Mitochondria moving in anterograde and retrograde directions appear as oblique lines. Scale bar: 5 µm. (E,F) Neither anterograde nor retrograde velocity of mitochondria is significantly different between any of the examined genotypes. Both anterograde and retrograde flux of mitochondria is reduced in mutant larvae (D42>khc^N262S^ and D42>khc^wt+N262S^) compared with controls (D42>w^1118^ and D42>khc^wt^). Statistical significance was determined using a Kruskal-Wallis H-test followed by a Dunn's test for comparisons between multiple groups. The standard error of the mean (s.e.m) is shown as a box, the standard deviation (s.d.) as a black line. * p<0.05; ** p<0.01. (See also Videos S4, S5, S6, S7).

We were thus interested in performing *in vivo* imaging to address the effects of the expression of Khc^wt+N262S^ on anterograde and retrograde cargo transport in motoneurons of the *Drosophila in vivo* model at a time point at which behavioral impairments can be observed. Data obtained in Khc^wt+N262S^-expressing larvae are therefore of particular importance. These larvae allowed us to quantify, for the first time, the effects of expression of mutated Khc at physiologically relevant ratios on axonal transport in the context of an intact nerve. In Khc^wt+N262S^-expressing larvae, stochastically more than 25% of the Khc motors are expected to be Khc^wt^-homodimers. We did not observe a significant change in the velocity of mitochondria in either direction in any of the investigated genotypes. We did, however, detect a strong reduction in both anterograde and retrograde flux in both Khc^N262S^ and Khc^wt+N262S^-expressing larvae. Khc^wt^-expressing larvae did not show any changes (Figure 5A–5F; Videos S4, S5, S6, S7).

**Figure 6:**
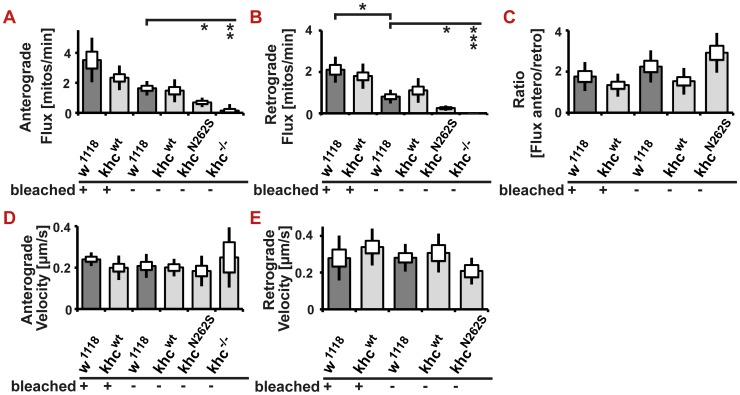
*In vivo* analysis of axonal transport in non-bleached nerves. (A–E) Analysis of mitochondrial transport in segmental nerves of *Drosophila* larvae. A segment of a nerve was—as indicated in the figure—either bleached to allow for better visualization of moving mitochondria, or was imaged in its native state. In all observed examples, the observed flux in bleached nerves was higher than in corresponding non-bleached samples. This reduction was significant, however, in only one instance (retrograde flux: D42>w^1118^). No effect of bleaching on transport velocities was observed. (A,B) Both anterograde and retrograde flux of mitochondria is reduced in khc mutant larvae (khc^−/−^) and in larvae expressing mutant Khc (D42>khc^N262S^) compared with control larvae. (C) The ratio of anterograde to retrograde flux has a tendency to be higher in the mutant (D42>khc^N262S^) than in the controls. (D,E) There is no significant difference in velocity between all genotypes. Statistical significance was determined using a Kruskal-Wallis H-test followed by a Dunn's test for comparisons between multiple groups. The standard error of the mean (s.e.m.) is shown as a box, the standard deviation (s.d.) as a black line. * p<0.05; ** p<0.01; *** p<0.001.

Ebbing and colleagues [7] reported that *in vitro* velocities of purified KIF5A constructs were reduced more than two-fold upon mixing wild-type and N256S-mutant kinesin at a stoichiometric ratio of 1∶1. The authors further assumed that kinesin cargo vesicles are moved by 5 to 8 motors [7]. Under these conditions, each organelle is expected to have a high probability of being attached to at least one mutant motor, leading to slower motility and shorter run lengths. The fact that we did not observe slower mitochondria suggests that the assumptions used to extrapolate single-molecule measurements to organelle transport in a cellular environment might be oversimplified. Alternatively, the experimental approach chosen to measure transport *in vivo* might be flawed. Bleaching is routinely used to quantify mitochondrial transport in *Drosophila* [12], [18]. To exclude an influence of the bleaching procedure on our results, we sought to compare flux and velocities obtained before and after bleaching. We are not aware of a study that experimentally validates that transport velocities are not affected by the bleaching procedure. Theoretically, slow mitochondria might not enter the bleached region during the analyzed time interval. Thus, they might be excluded from the analysis, resulting in a biased analysis due to an inappropriate selection of fast-moving mitochondria. By comparing the mitochondrial flux in bleached and in non-bleached nerve segments, we could show that bleaching has an effect on flux rates; a higher flux is observed when analysis is performed after bleaching (Figure 6A, 6B). This observation is best explained by the fact that bleaching allows for better visualization of moving mitochondria, which are less likely to be obscured after stationary mitochondria have been bleached. Both anterograde and retrograde flux is affected to the same degree by the method chosen. Thus, the ratio of retrograde to anterograde transport flux is not affected by the experimental procedure (Figure 6C). We observed no effect of bleaching on transport velocities (Figure 6D, 6E). To further confirm our results, we additionally performed a comparison of transport velocities obtained from two non-bleached control genotypes (D42>w^1118^; D42>Khc^wt^) and two non-bleached mutant genotypes (D42>Khc^N262S^; khc^−/−^). No significant reduction of anterograde or retrograde transport velocities was detected in any of the investigated phenotypes (Figure 6D, 6E). As no effect of bleaching on velocities could be observed, we suggest the use of bleaching when quantifying mitochondrial transport in *Drosophila* larvae.

**Figure 7:**
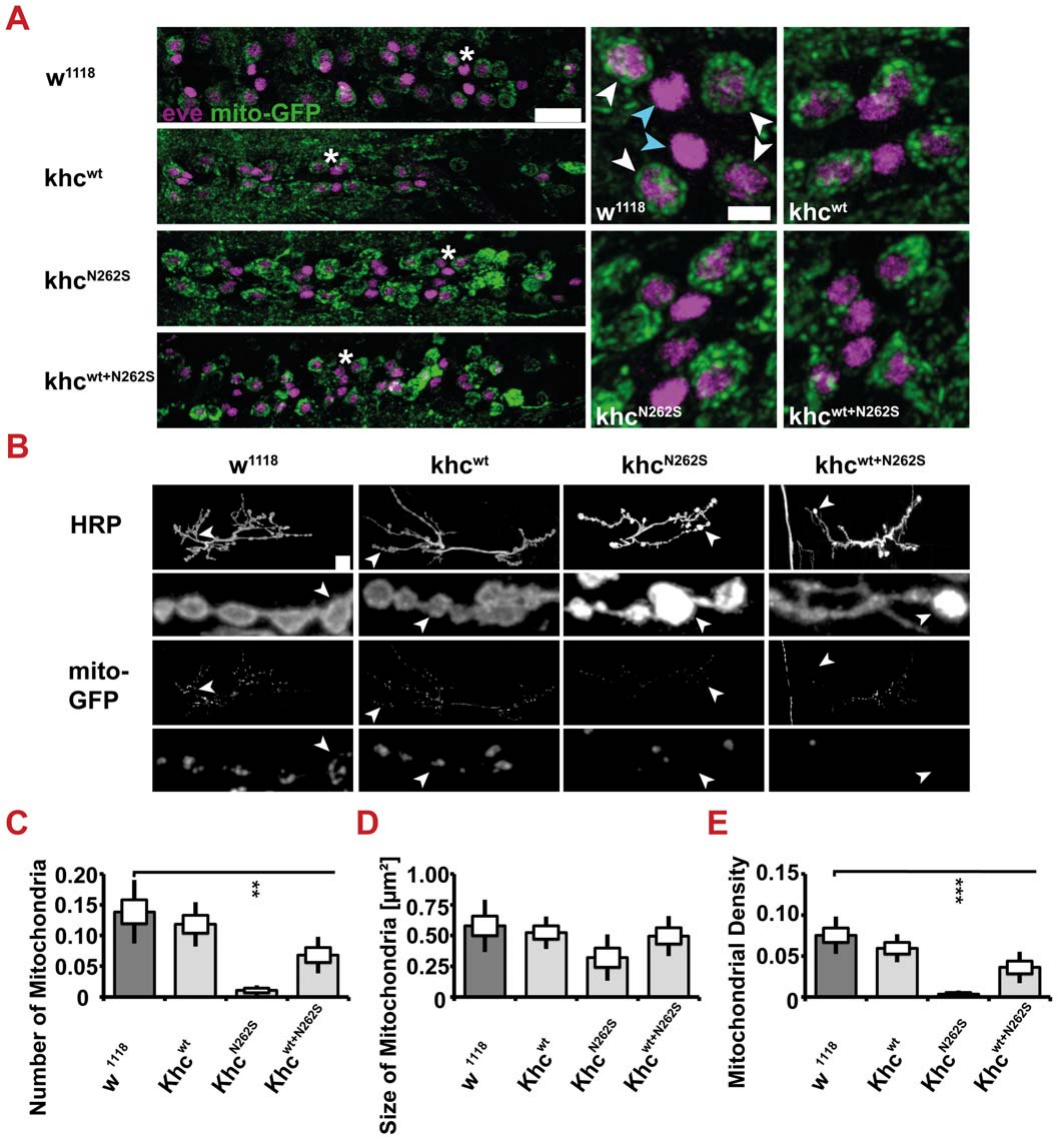
Mitochondrial content. Mitochondrial content was scored by immunofluorescent staining of larvae expressing mito-GFP in motoneurons. D42>w^1118^ or D42>khc^wt^ larvae were used as controls for the two mutant phenotypes D42>khc^N262S^ and D42>khc^wt+N262S^. (A) Confocal images of immunofluorescent staining of the ventral nerve cord (VNC) of mid-third-instar larvae. Staining for Even-skipped (eve, mangenta) was used to visualize the RP2 and aCC motoneuron (white arrow heads). The medially located interneurons (cyan arrowheads) are negative for mito-GFP (green). No reduction on mitochondrial content was observed in the two mutant phenotypes. Scale bar: 20 µm, and 5 µm in right panels. The star indicates the segment in the left panel that was used for the right panels. (B) Confocal images of immunofluorescent staining showing NMJ 6/7, segment A2 of mid-third-instar *Drosophila* larvae. In mutant larvae the mitochondrial content at the NMJ was lowered compared to the control larvae. An anti-HRP immunofluorescent staining was used to visualize neuronal membranes. Scale bar: 10 µm. The arrowhead indicates the section in the upper panel that was used for the enlargement shown in the lower panel. (C–E) Quantification of the mitochondrial number (C), size (D) and density (E) at NMJ 6/7, segment A2 of mid-third-instar *Drosophila* larvae. Mitochondrial Density is the mitochondrial Area Fraction relative to the NMJ size as quantified by the HRP staining. Statistical significance was determined using a Kruskal-Wallis H-test followed by a Dunn's test for comparisons between multiple groups. ** p<0.01, *** p<0.001. The standard error of the mean (s.e.m.) is shown as a box, the standard deviation (s.d.) as a black line.

We were next interested in further investigating the cause of the reduced mitochondria flux. The reduced flux might be directly caused by impairments in axonal transport or by depletion of mitochondria in the cell body or near synapses. No obvious reduction in mitochondrial abundance in motoneuron cell bodies was detected (Figure 7A). Thus, the observed reduction in anterograde flux is likely caused by impaired transport of mitochondria.

Although the size of mitochondria was not affected by expression of mutated Khc, a strong reduction in the number and density of mitochondria at neuromuscular junctions (NMJs) 6/7 in segment 2 was detected in Khc^N262S^-expressing larvae (Figure 7B–7E). A trend toward a reduced mitochondrial number (p = 0.07) and density (p = 0.08) in Khc^wt+N262S^-expressing larvae was observed (Figure 7C–7E). Thus, reductions in the retrograde flux might be the result of impaired retrograde axonal transport or of reduced abundance of mitochondria at the synapse, or a combination of both effects.

### Expression of Khc^N262S^ causes length-dependent synaptic defects at the neuromuscular junction

**Figure 8:**
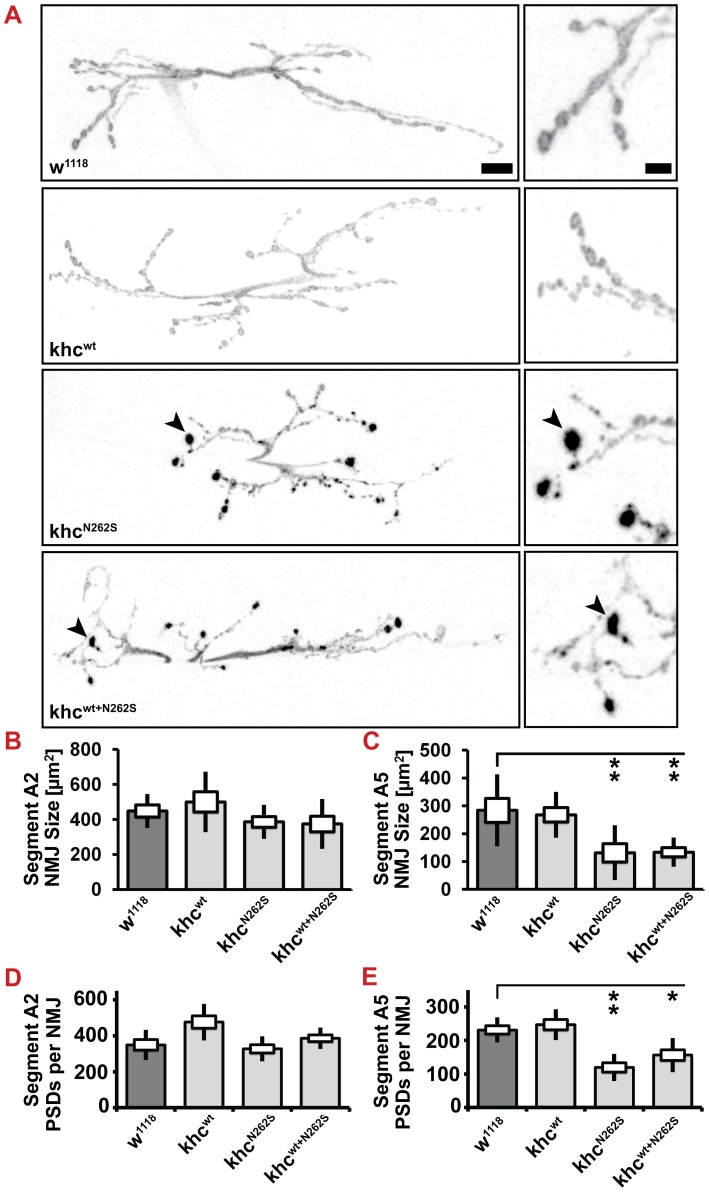
Neuromuscular morphology. (A) Confocal images of anti-HRP immunofluorescent staining showing NMJ 6/7, segment A2 of mid-third-instar *Drosophila* larvae. D42>w^1118^ or D42>khc^wt^ larvae were used as controls for the two mutant phenotypes D42>khc^N262S^ and D42>khc^wt+N262S^. Larvae were raised at 29°C. Frequently, boutons with abnormal anti-HRP accumulation (A, arrowheads) are observed in Khc^N262S^-expressing larvae. Scale bars: 10 µm, right panels 4 µm. (B, C) Quantification of NMJ area. Analysis was performed in both (B) the anterior segment A2 and (C) the posterior segment A5. (D, E) Quantification of synapse numbers (glutamate receptor fields) in segments A2 and A5. n = 8–10 NMJs per genotype and per segment were used for B–E. A one-way ANOVA followed by a Tukey-Kramer post-test was used for (B,C). Statistical significance in (D,E) was determined using a Kruskal-Wallis H-test followed by a Dunn's test for comparisons between multiple groups. * p<0.05; ** p<0.01. The standard error of the mean (s.e.m.) is shown as a box, the standard deviation (s.d.) as a black line.

Next, we were interested in studying the structure and function of NMJs in more detail. Behavioral experiments in Khc^wt+N262S^-expressing larvae and data obtained in *khc* null mutants [13] predict strong defects at posterior segments, whereas anterior segments should be less affected. Quantification of both the NMJ area and the synapse number (postsynaptic glutamate receptor fields) revealed that this is indeed the case (Figure 8A–8E). Overexpression of Khc^N262S^ or Khc^wt+N262S^ led to a strong reduction in the area of NMJs 6/7 in segment A5 but not in segment A2 (Figure 8A–8C). Affected NMJs are furthermore characterized by inhomogeneity in anti-HRP staining (Figure 8A, arrowheads). No significant reduction in the number of synapses was detected in anterior segment A2 (Figure 8D). Overexpression of Khc^N262S^ or Khc^wt+N262S^ caused a significant reduction, however, in the number of synapses (Figure 8E) in posterior segment A5.

**Figure 9:**
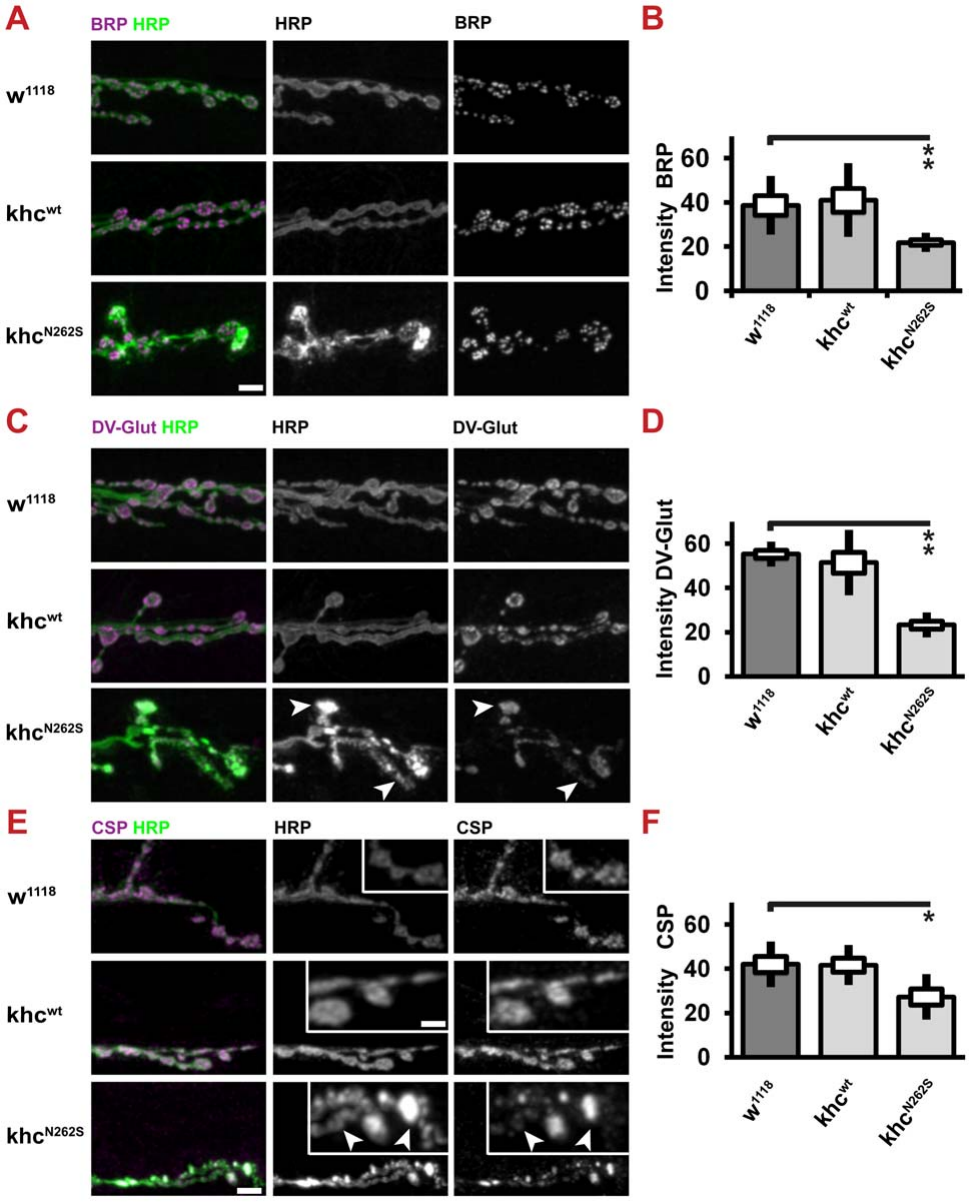
Expression of Khc^N262S^ causes length-dependent synaptic defects at the neuromuscular junction. Confocal images and quantification of immunofluorescent staining of synaptic marker proteins. NMJs 6/7, segment A5 of mid-third-instar *Drosophila* larva, were selected for analysis. D42>w^1118^ and D42>khc^wt^ larvae were used as controls for the mutant phenotype D42>khc^N262S^. All three selected synaptic marker proteins were reduced in abundance at the NMJs of khc^N262S^-expressing larvae. Larvae were raised at 29°C. (A,B) The active zone protein Brp, as well as the synaptic vesicle proteins (C,D) DV-Glut and (E,F) CSP, were selected as synaptic marker proteins. (A,C,E) Confocal images revealed that DV-Glut and CSP abundance is increased in dystrophic boutons (C,E, arrows) and reduced elsewhere (C,E, arrowheads) at NMJs of D42>khc^N262S^ larvae. Scale bar: 5 µm; inset 2.5 µm. (B,D,F) For quantification, n = 8–10 NMJs were analyzed per genotype. Statistical significance was determined using a Kruskal-Wallis H-test followed by a Dunn's test for comparisons between multiple groups. The standard error of the mean (s.e.m.) is shown as a box, the standard deviation (s.d.) as a black line. * p<0.05; ** p<0.01.

We next sought to address whether reduced axonal transport does limit the supply of NMJs with SVs and active zone proteins. To this aim, we used CSP and DV-Glut as markers for SVs and Brp as a marker for AZs. All three proteins are present in axonal swellings (Figure 3B, 3D, 3H). We could confirm that the abundance of both AZ (Figure 9A, 9B) and SV (Figure 9C–9F) proteins is reduced at the NMJ. SV proteins are inhomogenously distributed in Khc^N262S^-expressing larvae. While few boutons stain intensively for CSP and DV-Glut (Figure 9C, 9E arrowheads), other boutons display a weaker staining intensity (Figure 9C, 9E arrows). There is a strong correlation between the inhomogeneity observed in the staining for HRP and SV proteins. This inhomogenous distribution might resemble defects in the delivery of SV, in endo-/exocytosis, or in membrane trafficking.

**Figure 10:**
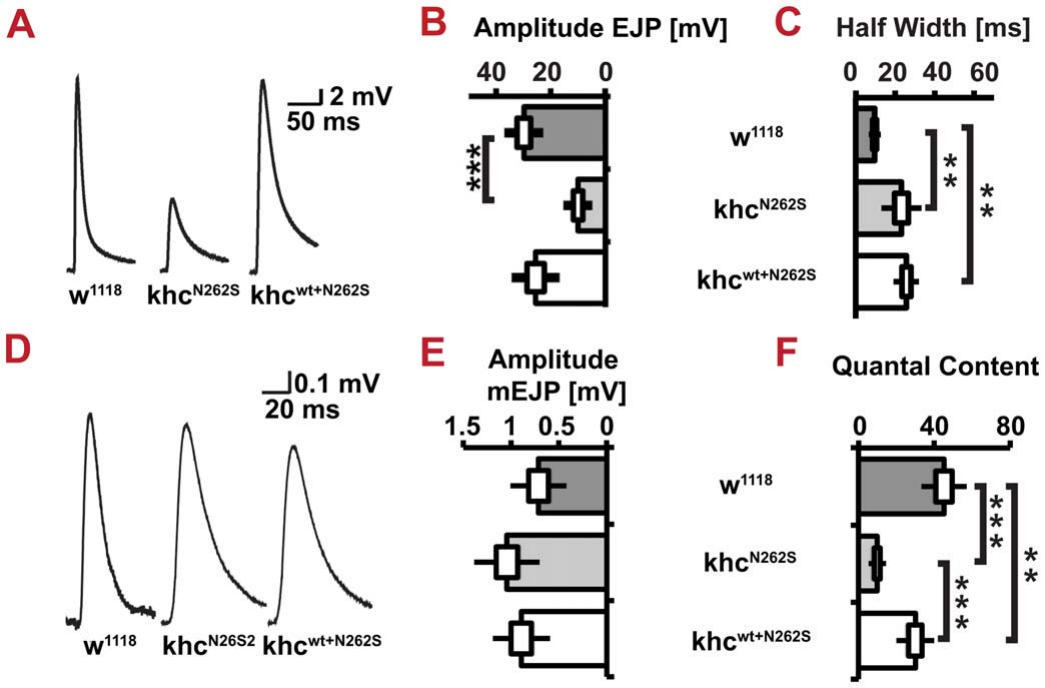
Neuromuscular function. Evoked junctional potentials (EJPs), the half width of EJPs and mini excitatory junctional potentials (mEJPs) were determined in D42>w^1118^, D42>khc^N262S^ and D42>khc^wt+N262S^ larvae. (A) Averaged traces of EJPs. Stimulation artifacts were removed. (B) Quantification of averaged EJP size. (C) Half width of EJPs. (D) Averaged amplitudes of mEJPs from control and mutant larvae. (E) Quantification of averaged mEJP size. (F) Quantification of the number of vesicles released per action potential. Statistics n = 8–9 larvae per genotype for B,C,E,F. One-way ANOVA followed by Tukey-Kramer post-test ** p<0.01; *** p<0.001. The standard error of the mean (s.e.m) is shown as a box, the standard deviation (s.d.) as a black line.

Thus, we were interested in addressing functional impairments of the NMJ in detail. To this aim, we recorded postsynaptic potentials by using current clamp recordings at muscle 6 in segment A4. The evoked excitatory junction potentials (eEJPs) of Khc^N262S^-expressing larvae were drastically decreased in amplitude and had an increase in the half-width time (Figure 10A, 10B, 10C). The increased half-widths in Khc^N262S^- and Khc^wt+N262S^-expressing larvae might be caused by impairments in the synchronization of vesicle fusion with the arrival and spread of the action potential to all release sites [23]. Loss of Brp - an active zone protein that has been shown to be important for establishing close proximity between SV and release sites - leads, in like manner, to an increase in the half-width of evoked excitatory junctional currents [23].

In contrast to eEJPs, the amplitude of miniature excitatory junction potentials (mEJPs) in response to single, spontaneous vesicle fusion events was slightly - but not significantly – increased in both Khc^N262S^- and Khc^wt+N262S^-expressing larvae (Figure 10D, 10E). This might represent a postsynaptic compensation presynaptic defect. Indeed, although there was no significant difference in eEJPs between Khc^wt+N262S^-expressing larvae and controls, the former group revealed a significant reduction in quantal content (Figure 10F). As quantal content is a measure of the number of vesicles released per presynaptic action potential, it is better suited for characterizing presynaptic defects than eEJP size.

### Degeneration but no motoneuron cell death was observed after expression of Khc^N262S^

**Figure 11:**
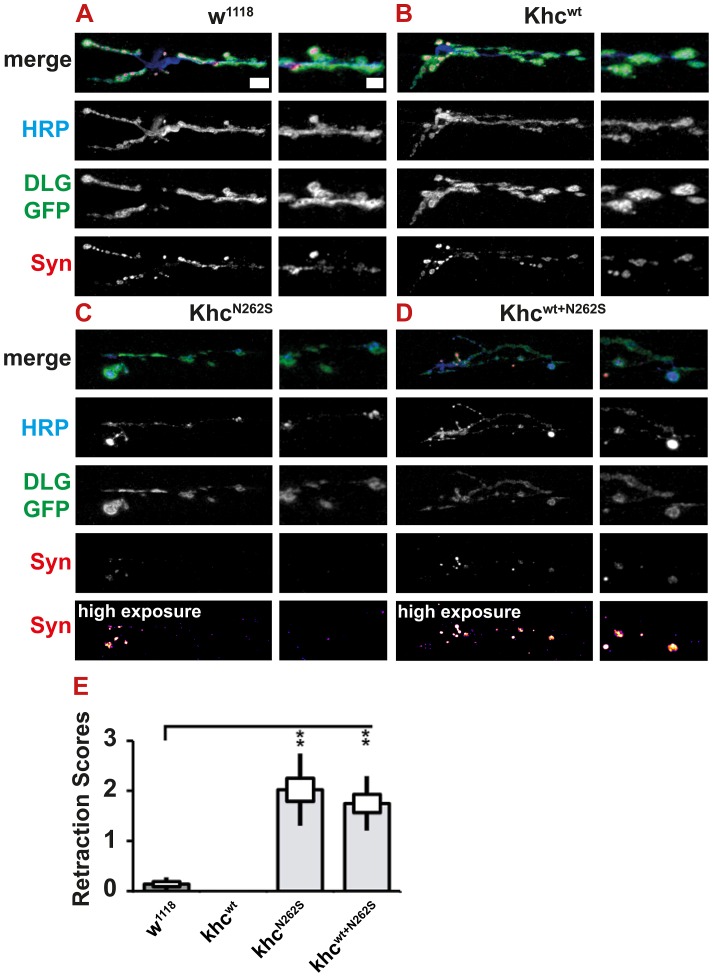
Light microscopic analysis of NMJ degeneration. Degeneration was revealed and scored by immunofluorescent staining. All larvae carried one copy of the motoneuron-specific driver D42-Gal4 and were raised at 29°C. (A–D) Confocal images of immunofluorescent staining showing NMJs 6/7, segment A5 of mid-third-instar *Drosophila* larvae. To visualize the subsynaptic reticulum, we used a GFP insertion in the discs-large locus. Neuronal membranes were visualized with an antibody against horseradish peroxidase (HRP). Synaptic vesicles were stained using m-α-Synapsin (Syn) antibody. For Khc^N262S^- and Khc^wt+N262S^-expressing larvae, which showed a strong reduction in Syn intensity, an additional, false-colored panel (high exposure) is shown. In this panel, the brightness was adjusted for better visibility of weak signals. Scale bar: 10 µm; right panels 5 µm. Genotypes: (A) D42>w^1118^; (B) D42>Khc^wt^; (C) D42>Khc^N262S^; (D) D42>Khc^wt+N262S^. (E) To integrate the frequency of retractions, we used a neurodegenerative scoring system to combine the occurrence of dystrophic boutons and minor pathological alterations at the NMJs into a single measure for the degree of pathological alterations (for details see Figure S1A–S1F). Using this scoring system, we detected a significant degree of neurodegenerative alterations in larvae expressing Khc^N262S^ either alone or in combination with Khc^wt^. Statistical significance was determined using a Kruskal-Wallis H-test followed by a Dunn's test for comparisons between multiple groups. The standard error of the mean (s.e.m.) is shown as a box, the standard deviation (s.d.) as a black line. ** p<0.01.

As HSP is a neurodegenerative disorder characterized by distal axonopathy, we were interested in whether we could observe any signs of synapse degeneration in our models. Both Khc^N262S^- and Khc^wt+N262S^-expressing larvae showed pathological alterations in neuronal membrane organization (Figure 8A; Figure 9A, 9C, 9E), as well as reduced abundance and altered distribution of SV proteins (Figure 9C, 9E; Figure 11A–11D). However, typically the complete absence of the SV protein synapsin from parts of the NMJ was not detected in Khc^N262S^-expressing larvae (Figure 9C, 9E; Figure 11C, 11D). These data are consistent with the reduction in abundance and inhomogeneous distribution of CSP and DV-Glut (Figure 9C, 9E) observed in Khc^N262S^-expressing larvae. Synaptic footprints [31], areas of the NMJ that are, after a retraction of the nerve, positive for postsynaptic marker proteins (Dlg or GluRIIC), but negative for presynaptic marker proteins, are commonly scored by using either synapsin [31]–[33] or Brp [34] as a presynaptic marker. The absence of a single presynaptic marker protein is a clear indication of pathological alterations at the NMJ. It is not sufficient, however, to prove that a nerve ending has retracted. We thus defined only the simultaneous absence of an SV marker (synapsin) and of the presynaptic membrane (HRP) as a retraction event. Using this more conservative assay, retraction was seldom detected. Dystrophic boutons characterized by a strong reduction in the intensity of SVs, in combination with an inhomogeneous HRP signal, were, however, frequently detected in animals expressing mutant Khc. To quantify the degree of neurodegenerative pathological alterations at the NMJs, we used a scoring system that assessed the frequency of retractions, the occurrence of dystrophic boutons, and the presence of minor pathological alterations such as weaker staining for SV (compare Figure S1A–S1F and Text S1). Using this scoring system, we detected a significant degree of neurodegenerative alterations in larvae expressing mutant Khc (Figure 11E).

**Figure 12:**
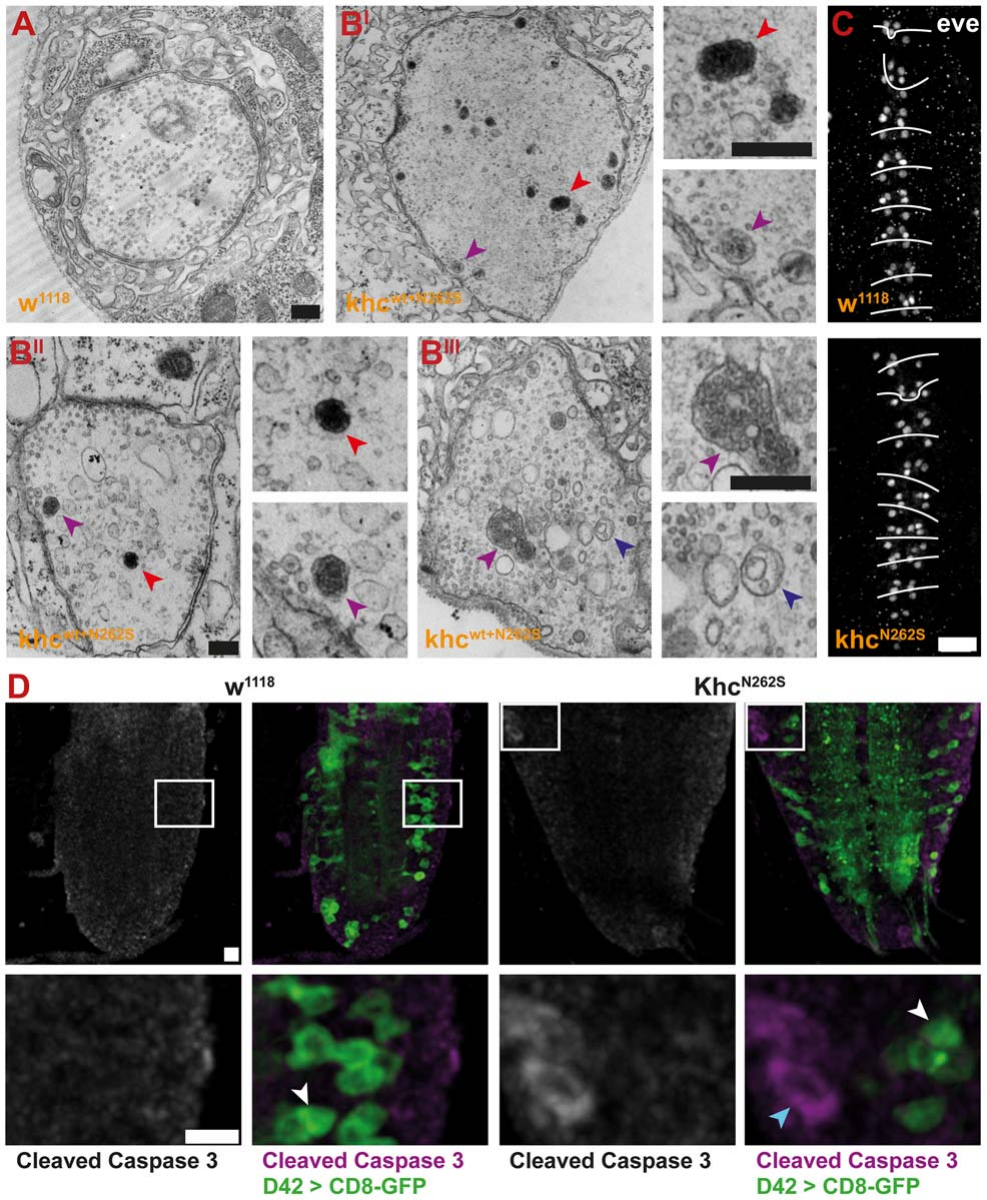
Analysis of NMJ degeneration and apoptosis. Degeneration was assessed by immunofluorescent staining and electron microscopy. All larvae carried one copy of the motoneuron-specific driver D42-Gal4 and were raised at 29°C. (A–B^III^) Electron micrographs of NMJ 4, segment A4, of mid-third-instar D42>w^1118^ and D42>Khc^wt+N262S^ larvae. Presynaptic boutons of D42>Khc^wt+N262S^ larvae contain multivesicular bodies (purple arrowheads), prelysosomal vacuoles (red arrowheads), and nascent autophagosomes (dark blue arrowheads). Scale bars: 100 nm. (C,D) Confocal images of immunofluorescent staining of the ventral nerve cord (VNC) of mid-third-instar larvae. (C) Staining for Even-skipped (eve) was used to score for loss of the RP2 and aCC motoneurons of D42>Khc^N262S^ and D42>w^1118^ larvae. Scale bar: 20 µm. (See also Figure S1G.) (D) A Cleaved Caspase-3 Antibody (magenta) was used to score for apoptosis. Motor neurons were visualized by expression of CD8-GFP (green) in D42>Khc^N262S^ and D42>w^1118^ larvae. Only very few apoptotic cells were detected (cyan arrowhead). No apoptosis in motoneurons (white arrowheads) was detected. Scale bar: 10 µm, and 10 µm in lower panels.

A strong HRP staining at a subset of boutons at the NMJs of larvae expressing mutant Khc suggested the local accumulation of membrane rich organelles. As autolysosomal organelles were detected in axonal swellings, we sought to address whether the observed strong HRP signal might be indicative of increased autophagy at the NMJ. Indeed, ultrastructural analysis of larvae overexpressing Khc^wt+N262S^ indicated that PLVs (red arrowhead), autophagosomes (dark blue arrowhead), and multivesicular bodies (purple arrowhead) are frequently present in mutant but not in control NMJs (Figure 12A and 12B^I^–12B^III^).

Next, we questioned whether the observed degeneration represents a classic distal synaptopathy or whether it is preceded by the loss of the motoneuron cell body. To this aim, we analyzed motoneuron cell bodies in 4-day-old early-mid L3 larvae, the stage at which degeneration was observed at the NMJ. No substantial motoneuron loss was detected in the subset of motoneurons positive for eve in Khc^N262S^-expressing larvae when compared with the control group (Figure 12C). To monitor cell death in all motoneurons we used an antibody that allows visualizing the activation of the putative initiator caspase DRONC [35]. Using this antibody, which is commonly used to quantify dying cells in *Drosophila* (for review see [35]), we found no evidence for increased apoptosis of motoneurons in mid L3 Khc^N262S^-expressing larvae (Figure 12D).

The fact that we did not observe substantial motoneuron cell loss in mid-L3 Khc^N262S^-expressing larvae, a stage when degeneration of synapses was already pronounced, is consistent with the concept that HSP is primarily caused by synaptopathy and axonopathy, whereas motoneuron cell loss is neither causative for HSP nor an early feature of the pathological process.

## Discussion

### Haploinsuffiency is an unlikely cause for SPG10

Human genetics suggest that autosomal-dominant SPG10 is not caused by haploinsuffiency. The above presented *Drosophila* model further supports this hypothesis. While no difference in the locomotion speed between wild-type larvae and larvae lacking one copy of *khc* were observed, larvae ectopically expressing Khc^N262S^ were severely impaired. The observed behavioral impairments are both qualitatively and quantitatively similar to impairments observed in *khc* null larvae.

These results are consistent with Khc^N262S^ acting as an antimorph or a neomorph. Antimorphic mutations act in opposition to the normal gene function. Thus, they are also referred to as dominant negative mutations [36]. The phenotypic severity of an antimorphic mutation will be decreased by increasing wild-type gene dosage [36]. A neomorphic mutation leads to a change in the nature of the gene resulting in a dominant gain of function. This function, which is not produced to a relevant degree by the native gene, is often toxic. Increasing wild-type gene dosage will not reduce the phenotypic severity of a neomorphic mutation, as the newly gained function is by definition different from the normal gene function [36].

Animals ectopically expressing Khc^N262S^ alone were more severely affected than animals expressing Khc^N262S^ in combination with an additional copy of Khc^wt^. We thus conclude that Khc^N262S^ is an antimorphic mutation: Khc^N262S^ causes loss of kinesin-1 function via a dominant-negative mechanism.

We propose that all observed pathological changes are downstream of a loss of kinesin-1 function and cannot be attributed to more generalized toxicity of mutant Khc or kinesin-1 complexes containing mutant Khc. In human patients, 25% of the Khc motors are expected to be Khc^wt^-homodimers. Our results suggest that these should be capable of transporting synaptic cargo.

### Coordination of transport

Two models have been proposed to explain how molecular motors coordinate cargo transport (for review, see [37]). The tug-of-war model assumes that the direction of movement is the result of the dynamic competition of opposing motors. The simultaneous action of two motors thus exerts a stretching force on the particle. Loss of motors responsible for transport in one direction should increase transport velocities in the opposite direction. While the direct opposing action of dynein and the *Dictyostelium* kinsesin-3 family member Unc-104 could be experimentally validated [38], most frequently, the loss of motors responsible for transport in one direction leads to transport disturbances in both directions [30], [39]. This is best explained by a model in which the activity of opposing motors is controlled by coordination complexes that are regulated such that only one set of motors is active at any given time. These two models are, however, not mutually exclusive. Both models offer distinct advantages for molecular motors. Ideally, coordinated action of opposing motors might allow for faster, more energy-efficient transport, but simultaneous binding of opposing motors has been proposed to decrease the probability of detachment from microtubules, resulting in increased processivity of movement [40]–[42]. Thus, it is most probable that in most cellular environments cargo is transported by the coordinated, simultaneous action of opposing motors. The exact balance between coordinated inactivation of opposing motors and their active role as a stabilizing “dragging force” might vary for different cargos, developmental time points, stages of disease progression, and distinct cell types. Thus, it is of no surprise that studies investigating mutation in molecular motors performed in different model systems lead to seemingly opposing results.

In *Drosophila*, mitochondrial transport velocities are affected neither by the deletion of *khc* [12] nor by overexpression of Khc^N256S^. These observations are in accordance with data obtained by measuring neurofilament transport in cultured mouse cortical neurons ectopically expressing KIF5A^N256S^ [29]. Yet how can these results be reconciled with the observation that maximum and average velocities of mitochondrial [29] and neurofilament [28] transport were reduced in neurons isolated from KIF5A^−/−^ mouse embryos? The loss of KIF5A might be partially compensated by KIF5B or KIF5C. KIF5B and KIF5C might be less effective, however, in transporting mitochondria, thus reducing both average and maximum transport velocity. While no compensatory up-regulation of KIF5B or KIF5C was detected in KIF5A^−/−^ mice [30], indirect evidence nonetheless suggests that residual mitochondrial transport observed in KIF5A^−/−^ motoneurons might be driven by KIF5B or KIF5C. First, both mitochondria and neurofilaments are actively transported in the anterograde direction [28], [30]. Thus, they must be bound to an anterograde motor. Second, KIF5C and KIF5B have been shown to be important for mitochondrial transport [43]. In KIF5B^−/−^ cells, mitochondria are clustered around the nucleus rather than being appropriately dispersed throughout the cell. This defect can be rescued by ectopic expression of KIF5A, KIF5B, or KIF5C, highlighting the fact that any of these motors are capable of transporting mitochondria [43]. While the frequency of neurofilament transport is reduced by 75% in KIF5A^−/−^ neurons, KIF5C or KIF5B overexpression is sufficient to partially rescue this defect [28].

We thus propose that cellular quality control mechanisms ensure that cargos are equipped with a minimal number of molecular motors. Thus, obvious defects such as the genetic deletion of KIF5A are detected and partially compensated by targeting similar motors to cargos awaiting initiation of transport. Transport driven by these alternative motors might be slower and less efficient [28], [30]. Still, a basal cargo flux can be obtained despite the complete absence of one molecular motor type [28], [30]. Mutations in KIF5A that impact neither the stability of the protein nor its ability to interact with regulatory complexes might not be detected by this cellular quality control system. Thus, cargos might be loaded with defective motors that cause problems after initiation of transport. These secondary problems might include a drop of transport flux [28], [29]. The phenotypic strength of defects might therefore depend on the expression level as well as the nature of the mutation. Thus, observed defects might be either less severe (KIF5A^N256S^ [29]) or more severe (head-less KIF5A, [28]) than loss of KIF5A [28].

### Expression of mutant Khc results in distal synaptopathy

Khc^N262S^-expressing larvae exhibit the characteristics of classical distal degeneration. No human SPG10 autopsy reports have been published till date, but studies of SPG4 cases [1] suggest a “dying back” axonopathy as probable disease mechanism in HSP. Synapse and axon loss may therefore be a primary step in the pathophysiological manifestation of SPG10, while neuronal loss may occur only later.

Could protection of the cell bodies be used as a suitable treatment strategy for HSP? While little data is available on the treatment of HSP patients, treatment strategies for Amyotrophic lateral sclerosis (ALS), a clinically related disease, have been explored in more detail. The neurodegenerative disease ALS is characterized by the progressive loss of motoneuron in the brain and the spinal cord [44]. Pathological changes occur in ALS patients and animal models - similar to our observations in the *Drosophila* HSP model - first at the NMJ and are followed by axon and neuronal loss [45], [46].

Treatment strategies aiming at prevention of motoneuron loss lead only to limited success in ALS models and human patients [44]. Thus, treatment that aims at delaying motor impairments during the progression of ALS is currently favored [44]. We suggest that preservation of synapses and axons will be an important requirement for a successful therapeutic intervention of both HSP and ALS.

### Relevant cargo

Impaired neurofilament transport has been implicated in the pathogenesis of SPG10 [29]. The human genome contains 3 genes encoding the heavy chains of conventional kinesin: the neuronally expressed genes *KIF5A* and *KIF5C* and the ubiquitously expressed gene *KIF5B*. Neurofilaments are transported by KIF5A and KIF5C [28], [29]. Impairments in neurofilament transport due to impaired KIF5A function might thus explain why only mutations in KIF5A, but not KIF5B, have been identified as a genetic cause for HSP.

The *Drosophila* genome, on the other hand, does not contain a neurofilament gene [47]. Electron microscopic studies concluded that neurofilaments are absent in all arthropods [48] and yet ectopic expression of mutant Khc leads to the formation of axonal swellings and HSP-like pathological changes in *Drosophila*.

Conversely, the hypothesis that impairments in neurofilament transport represent an important cellular cause of HSP has been further supported by reports that swellings in SPG4 patients and the SPG4 mouse model were positive for neurofilaments [49].

These swellings also contained, however, multiple other cargos, including mitochondria, the amyloid precursor protein, tubulin, and tau [49]. In our study, both kinesin-1 and kinesin-3 cargos [24], as well as lysosomal and autophagic organelles, accumulated in swellings. This suggests that eventually all kinds of cargo might become trapped in axonal swellings.

Future studies involving the use of *in vivo* imaging in mouse and *Drosophila* models will be needed to shed light on the formation of swellings. It is therefore important to identify cargos that accumulate first within a swelling. The cargo, which causes the formation of the swellings, should accumulate prior to other cargos.

A more detailed understanding of the temporal sequence of cargo accumulation and an increase in the understanding of impaired neurofilament transport will be instrumental to further decipher common pathological hallmarks of HSP. It is to be hoped that this will aid in the design of successful treatment strategies.

## Materials and Methods

### Molecular biology

Site-directed mutagenesis was used to introduce the amino acid exchange N262S in a full-length wild-type *Drosophila khc* cDNA (SD02406) which was next inserted into a modified pUAST attB vector. Details are described in Text S2.

### Fly stocks

Flies were maintained at 25°C on standard fly medium seeded with yeast. For experiments, flies were raised at 18°C, 25°C, or 29°C. For fly strains used in this study, see Table S1. Transgenic stocks UAS-khc^wt^ and UAS-khc^N262S^ were created by BestGene using integrase mediated site-specific transgenesis at cytological position 86F (Fly strain BDSC 23648).

### Western blot

Proteins were extracted from whole fly heads using 65 mM Tris (pH 6.8), 5% (w/v) SDS, 1× Protease Inhibitor (Roche) buffer. Samples were separated on 7.5% SDS gels and transferred to nitrocellulose membranes. For antibodies used in this study, see Tables S2 and S3.

### Immunohistochemistry and microscopy

Third instar larvae were dissected in chilled Ca^2+^-free HL3 solution and fixed in 4% formaldehyde in PBS. For antibodies used in this study, see Tables S2 and S3. Larval preparations were mounted in Vectashield (Vector). Images were captured using a Zeiss LSM 710 confocal microscope with the following settings unless otherwise noted: Objective: 40× plan Apochromat, 1.3 N.A.; Voxel Size: 100 nm×100 nm×500 nm; pinhole: 1 AU, average: 2–4. Images used for illustration purposes were processed as follows: (1) A Gaussian filter (radius = 2) was applied to the raw data stack. Brightness and contrast were appropriately adjusted. The relevant slices of the modified stacks were maximum-projected. Projected images were scaled by 2, and gamma adjustment (gamma = 0.75) was applied. ImageJ 1.41o, 1.44p, 1.46r or 1.45s (US National Institutes of Health; http://rsb.info.nih.gov/ij/download.html) was used to process and analyze images. For quantification of Glutamate receptor fields, Delta 2D software (Decodon GmbH, Germany) was used.

### *In vivo* imaging

*In vivo* imaging was essentially performed as previously described [50]–[52], using a Zeiss LSM 710 confocal microscope equipped with a 40× Plan Apochromat Objective (1.3 N.A.). For better visualization of moving mitochondria, all mitochondria in a 20 µm segment of the nerve were bleached. This allowed for easy visualization of moving particles passing through the bleached region. Next, confocal Z-stacks (Z-planes: 10; voxel size: 100 nm×100 nm×1500 nm, pinhole: 1.6 AU, average: 2) were recorded at maximal speed corresponding to 100 stacks per 426 seconds. For details on the generation of kymographs, see Text S1.

### Electrophysiology

Third instar larvae were dissected, rinsed, and transferred into the recording chamber. A fixed-stage upright microscope (Model BX51WI with 40× water immersion lens; Olympus Optical, Tokyo, Japan) was used to visualize the nerve and the muscles. Intracellular current clamp recordings were performed in HL3 solution with 1 mM extracellular Ca^2+^ at 19°C. Evoked excitatory junction potentials (eEJPs) and spontaneous miniature excitatory junction potentials (mEJPs) were obtained from muscle 6 segment A4 with an Axoclamp 900A amplifier (Axon Instruments, Union City, CA), digitized (Digidata 1440A, Axon Instruments, Union City, CA), recorded at 10 kHz (pClamp 10, Axon Instruments, Union City, CA), and analyzed using AxoGraph X software. Sharp, bee-stinger-shaped glass microelectrodes filled with 3 M KCl and a resistance between 10 and 20 MΩ were used. Cells with resting potentials between −60 and −70 mV and input resistance >4 MΩ were selected for analysis.

For stimulation, the cut end of the segmental nerve was pulled into a fire-polished suction electrode (6–8 µm inner diameter), and brief (300 µs) depolarizing pulses were passed at 0.1 Hz (ISO-STIM 01D, NPI Electronics, Tamm, Germany, stimulus generator and isolation unit). The amplitude of the pulse was set to about 7V, which results in the stable recruitment of both innervating motoneurons. It corresponds to 1.5 times the amplitude needed to recruit both motoneurons innervating muscle 6. For each eEJP and mEJP average, 15 eEJPs and 120 s of mEJP recordings were used for subsequent analysis.

### Sample preparation for electron microscopy analysis of NMJs

Larval fillets were fixed with 4% PFA (in PBS) for 10 min at room temperature followed by fixation in 2.5% glutaraldehyde (in PBS) overnight at 4°C. Postfixation was done with 1% osmium tetroxide in 100 mM phosphate buffer, pH 7.2, for 1 h on ice. Larval fillets were rinsed with water, treated with 1% aqueous uranyl acetate (UA) for 1 h at 4°C, dehydrated through a graded series of ethanol concentrations, and stored in liquid Epon overnight. Next, larval fillets were pinned on a dissection pad. Muscles 4 of segment 4 were dissected with sharp insect pins, embedded in Epon, and polymerized for 48 h at 60°C. Ultrathin sections were stained with UA and lead citrate and viewed in a Philips CM10 electron microscope.

### Sample preparation for electron microscopy analysis of axons

Living L2 larvae were transferred to aluminum platelets with a 150 µm recess containing 1-hexadecene as an external nonpenetrating filler. The platelets were sandwiched with platelets that had no recess and cryofixed with a high-pressure freezer (Bal-Tec HPM 010, Balzers, Liechtenstein). Larvae were freed from external hexadecene under liquid nitrogen and then transferred to 2 ml microtubes with screw caps (Sarstedt, #72.694, Germany). As a freeze substitution medium, we used a 2% osmium tetroxide solution in anhydrous acetone, supplemented with 25 µl of 20% methanolic UA solution to give a final UA concentration of 0.5%. The freeze substitution was carried out in a Leica AFS-2 with the samples kept at −90°C for 27 h, −60°C for 6 h, and −40°C for 6 h. The temperature increase between steps was set to 10°C/h. At −40 h, glutaraldehyde from a 25% solution in water (EMS #16530, Electron Microscopy Sciences, Fort Washington, PA) was added to give a final concentration of 0.6% glutaraldehyde and 2% water. After 6 h at −40°C, the microtubes were placed on ice for another 1 h. Samples were then washed 3 times with acetone and infiltrated with Epon at room temperature in a series of increasing Epon concentrations in acetone (30%, 60%, 90%, 2× 100% Epon each for 1 h, with the second 100% Epon change continuing overnight on a rotating wheel). After embedding, the Epon samples were polymerized for 48 h at 60°C. Ultrathin sections were stained with UA and lead citrate and viewed in a Philips CM10 electron microscope. Micrographs were taken on EM-film (Maco ES 208, Hans O. Mahn GmbH & Co KG, Stapelfeld, Germany). Alternatively, sections were imaged by using a FEI-Tecnai Spirt, 120 kV electron microscope equipped with a Gatan USC 4000 camera.

### Resin sections

Thoraxes were dissected by careful removal of heads, wings, limbs, and abdomen parts, prefixed at 4°C overnight (4% PFA, 3% glutaraldehyde, 0.1% sodium cacodylate), and postfixed with 1% osmium tetroxide for 3 h at 4°C. Next, thoraxes were washed with 30%, 50%, 70%, and 100% ethanol for 10 min each followed by washing with 100% acetone and 3∶1 acetone∶Epon for 1 h. Thoraxes were then immersed in 1∶1 acetone∶Epon and 100% Epon for 24 h. The Epon was polymerized for 48 h at 60°C. Semi-thin sections (2 µm) were prepared on a Reichert-Jung Supercut 2050 microtome with glass knifes. The semi-thin sections were stained with Toluidine blue solution (0.5% Toluidine blue O [C.I 52040, Roth] in 1% [w/v] disodium tetraborate buffer) for 1 minute and then washed under running water. The semi-thin sections were documented on a Zeiss Imager.Z1m microscope by using a 20× Zeiss Neofluar, 0.5 N.A objective. All washing, fixation, or staining procedures were performed at room temperature unless otherwise noted.

### Survival assay

Flies were raised at 18°C. Offspring were collected on the day of eclosion and 15–20 male flies transferred to vials containing standard fly media. The flies were transferred to fresh fly media every 3 days. A Kaplan-Meier plot was used for depicting survival curves.

### Climbing assay

Flies were raised at 18°C. Emerged male flies were split into two batches of 50 flies within 24 h after eclosion. One batch of flies was raised at 18°C and the other at 29°C to induce the expression of either Khc^wt^ or Khc^N262S^ for 16 days. Motor function of 16-day-old flies was monitored by analyzing their ability to climb 6 cm at the wall of a vertical plastic tube within 15 s. Fifty flies from each genotype were analyzed. A successful trial was scored with 1 and a nonsuccessful trial with 0. Each fly was allowed to climb three times and the average climbing score per fly was calculated.

### Locomotion analysis

To monitor locomotion behavior, we placed individual larvae on a thin slice of apple juice agar. Locomotion was examined at 25°C at 70% humidity by using a DCM510 (ScopeTek, P.R. China) camera integrated in a custom-built stereomicroscope. Larval locomotion was recorded at a frame rate of 30 fps for 5 min. The videos were then converted into avi format by using a Prism Video Converter, v 1.61 (NCH Software Inc., Australia). Next, images were cropped and compressed by using VirtualDub 1.9.10 (http://www.virtualdub.org/).

To measure locomotion speed, we placed up to 200 larvae on a 15×15 cm agar plate and filmed for 10 min. Locomotion and size of the larvae were analyzed with the custom-built software *Animaltracer*. This software was developed by us on the basis of the MATHLAB software package Worm Tracker & Track Analyzer (Department of Molecular and Cellular Physiology at Stanford University) [53]. This algorithm can be divided into two parts, the larval tracker and the track analyzer. The larval tracker identifies and tracks individual larvae within a movie. The track analyzer analyzes the movies and returns the size and the velocity of single larvae. For comparative analysis of the different genotypes larvae within a certain size range were grouped. Average locomotion speed is calculated for this size group for every movie. Larvae that touched each other were automatically excluded from analysis. Larvae whose velocity was less than 10% of average velocity of the respective genotype and size group were likewise excluded from analysis. A minimum of six movies per genotype were analyzed. For all further statistical analysis, n was defined as the number of movies analyzed.

### Analysis in mouse embryonic motoneuron culture

*KIF5A^+/−^* mice were obtained from MMRRC (Mutant Mouse Regional Resource Centers, University of California, Davis, USA). Mouse embryonic motoneuron culture, staining of mitochondria, and time-lapse imaging were performed as previously described [30]. Flux of mitochondria was measured in a 20 µm segment of motoneuron axons.

The number of mitochondria passing two defined cross-sections, both in the anterograde and the retrograde direction, were counted in a time interval of 30 minutes. The flux is the average of these two measurements. Mitochondria that moved less than 5 µm within the 20 µm segment were classified as stationary. 18 *KIF5A^+/+^* and 23 *KIF5A^−/−^* axons from four independent experiments were analyzed.

All animal work in this study were approved by the German Government (Regierungspräsidium Tübingen) and the University of Tübingen.

### Statistical analysis

Statistical tests were performed with the software PAST.exe (http://folk.uio.no/ohammer/past/index.html) unless otherwise noted. Sample errors are given as standard deviation (s.d.) and standard error of the mean (s.e.m). Data were first tested for normality by using the Shapiro-Wilk test (α = 0.05). Normally distributed data were analyzed either by student's t-test (two groups) or by a one-way analysis of variance followed by a Tukey-Kramer post-test for comparing multiple groups. Non-normal distributed data were analyzed by using either a Mann-Whitney test (two groups) or a Kruskal-Wallis H-test followed by a Dunn's test for comparisons between multiple groups. Differences in survival were determined by the Mantel-Cox test using Prism. The p values obtained from the Mantel-Cox test were corrected for the total number of comparisons made. Statistical tests for analyzing axonal transport in KIF5A-deficient motor neurons were performed with IBM SPSS Statistics, Version 20. The following alpha levels were used for all tests: * p<0.05; ** p<0.01; *** p<0.001.

## Supporting Information

Figure S1 (Related to Figure 11.) Illustration of quantification of NMJ retraction. (A–E) Quantification of NMJ retraction. An additive scoring system was used to score degeneration (E). (A–D) Samples of NMJs with no (A, B) or varying severity of degeneration (C, D). Scale bars: 10 µm. (F) Quantification of motor neuron cell loss by staining for Even-skipped which marks the medially located motor neurons RP2 and aCC, as well as the pCC interneurons. For quantification only the motor neurons in the segments A1–A3 and A5–A7 were scored. Scale bars: 20 µm.
(TIF)

Table S1 *Drosophila* stocks used in this study.
(DOCX)

Table S2 Primary antibodies used for immunochemistry and western blot in this study.
(DOCX)

Table S3 Secondary antibodies used for immunochemistry and western blot in this study.
(DOCX)

Text S1 Supporting image quantification methods.
(DOCX)

Text S2 Supporting molecular biology methods.
(DOCX)

Video S1 (Related to Figure 1.) Locomotion of Khc^N262S^ expressing larvae. The locomotion of a 4-day-old L3 D42>Khc^N262S^ larva at 25°C. Movie is shown at 30 fps. The larva is almost completely paralyzed, it is unable to crawl but still able to move its head.
(AVI)

Video S2 (Related to Figure 1.) Locomotion of Khc^wt+N262S^ expressing larvae. The locomotion of a 4-day-old L3 D42>Khc^wt+N262S^ larva at 25°C. Movie is shown at 30 fps. The larva is only partially paralyzed and shows characteristic tail-flipping phenotype. The crawling speed of D42>Khc^wt+N262S^ and control larvae is however similar.
(AVI)

Video S3 (Related to Figure 1.) Locomotion of Khc^wt^ expressing larvae. The locomotion of a 4-day-old L3 D42>Khc^wt^ larva at 25°C. Movie is shown at 30 fps. The larva crawls normally.
(AVI)

Video S4 (Related to Figure 5.) *In vivo* imaging of mitochondrial transport in control larvae. *In vivo* imaging of axonal transport of mitochondria in control larvae (w^1118^). Movie is shown at 15 fps. Mitochondria were visualized by D42>mito-GFP expression at 29°C.
(AVI)

Video S5 (Related to Figure 5.) *In vivo* imaging of mitochondrial transport in Khc^wt^ larvae. *In vivo* imaging of axonal transport of mitochondria in Khc^wt^ expressing larvae. Movie is shown at 15 fps. Mitochondria were visualized by D42>mito-GFP expression at 29°C. No significant differences in velocity or flux of mitochondria between control and Khc^wt^ expressing larvae was observed.
(AVI)

Video S6 (Related to Figure 5.) *In vivo* imaging of mitochondrial transport in Khc^N262S^ larvae. *In vivo* imaging of axonal transport of mitochondria in Khc^262^ expressing larvae. Movie is shown at 15 fps. Mitochondria were visualized by D42>mito-GFP expression at 29°C. Flux but not velocity of mitochondria is strongly reduced both anterogradely and retrogradly when compared to w^1118^ and Khc^wt^ expressing larvae.
(AVI)

Video S7 (Related to Figure 5.) *In vivo* imaging of mitochondrial transport in Khc^wt+N262S^ larvae. *In vivo* imaging of axonal transport of mitochondria in Khc^wt+N262S^ expressing larvae. Movie is shown at 15 fps. Mitochondria were visualized by D42>mito-GFP expression at 29°C. Flux but not velocity of mitochondria is strongly reduced both anterogradely and retrogradly when compared to w^1118^ and Khc^wt^ expressing larvae.
(AVI)
