# Correction: Spastic Paraplegia Mutation N256S in the Neuronal Microtubule Motor KIF5A Disrupts Axonal Transport in a *Drosophila* HSP Model

**DOI:** 10.1371/annotation/2f7219d5-14a3-4df8-a9ff-1a1f11251b27

**Published:** 2013-04-15

**Authors:** Petra Füger, Vrinda Sreekumar, Rebecca Schüle, Jeannine V. Kern, Doychin T. Stanchev, Carola D. Schneider, Kathrin N. Karle, Katharina J. Daub, Vera K. Siegert, Matthias Flötenmeyer, Heinz Schwarz, Ludger Schöls, Tobias M. Rasse

An error was introduced in the funding information. Correction text below:

An error was introduced in the funding information during the preparation of this article for publication. The correct funding information is as follows: This work was supported by a grant from the Hertie Foundation to RS and TMR, by grants from the German Competence Network 'Degenerative Dementias' (01GI1005B) and the Fritz Thyssen Foundation (grant 10.12.1.192) to TMR and a grant from the Center for Clinical Research (IKFZ) Tübingen (grant 170-0-0) to RS. The funders had no role in study design, data collection and analysis, decision to publish, or preparation of the manuscript.

