# Supplementary figures and images for: Spastic Paraplegia Mutation N256S in the Neuronal Microtubule Motor KIF5A Disrupts Axonal Transport in a Drosophila HSP Model
Source: PLoS Genet. 2012 Nov 29;8(11):e1003066. doi: 10.1371/journal.pgen.1003066 (PMC3510046; doi:10.1371/journal.pgen.1003066)

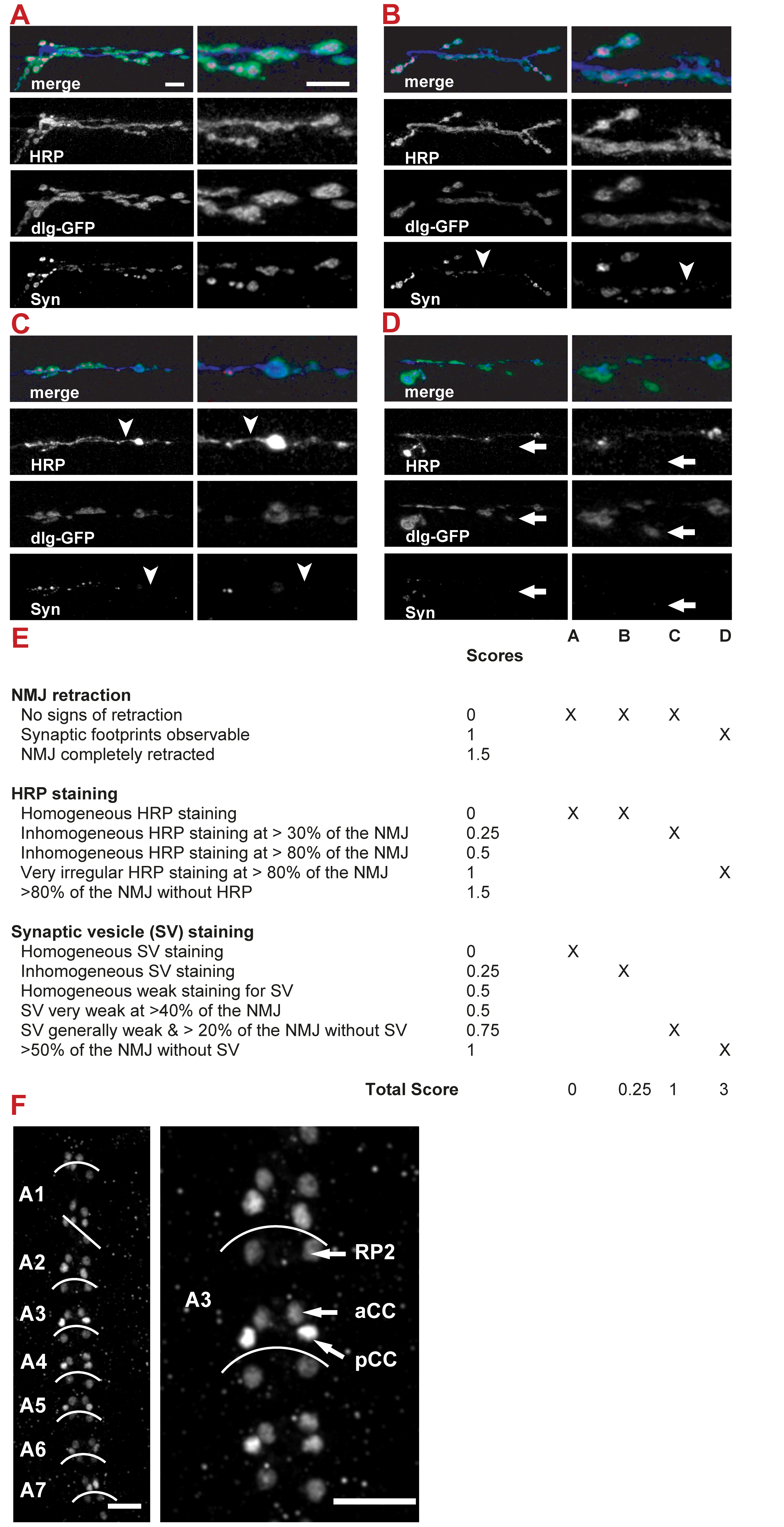

Supplement: Figure S1 — (Related to Figure 11.) Illustration of quantification of NMJ retraction. (A–E) Quantification of NMJ retraction. An additive scoring system was used to score degeneration (E). (A–D) Samples of NMJs with no (A, B) or varying severity of degeneration (C, D). Scale bars: 10 µm. (F) Quantification of motor neuron cell loss by staining for Even-skipped which marks the medially located motor neurons RP2 and aCC, as well as the pCC interneurons. For quantification only the motor neurons in the segments A1–A3 and A5–A7 were scored. Scale bars: 20 µm. (TIF) [file pgen.1003066.s001.tif]
