# Supplementary material for: Spastic Paraplegia Mutation N256S in the Neuronal Microtubule Motor KIF5A Disrupts Axonal Transport in a Drosophila HSP Model
Source: PLoS Genet. 2012 Nov 29;8(11):e1003066. doi: 10.1371/journal.pgen.1003066 (PMC3510046; doi:10.1371/journal.pgen.1003066)
Supplement: Table S1 — Drosophila stocks used in this study. (DOCX) [file pgen.1003066.s002.docx]

**Table S1. *Drosophila* stocks used in this study**

| Allele | Chromosome | Donor/  BDSC number | Reference |
| --- | --- | --- | --- |
| *khc^8^* | II | BL 1607 | [[1](#_ENREF_1)] |
| *khc^13314^* | II | BL 11084 | [[2](#_ENREF_2)] |
| Gal4-Driver | Chromosome |  | Reference |
| D42-Gal4 | III | BL 8816 | [[3](#_ENREF_3)] |
| elav^C155^-Gal4 | X | BL 458 | [[4](#_ENREF_4)] |
| tub-gal80^ts^ | X | BL 7016 | [[5](#_ENREF_5)] |
| UAS-Construct | Chromosome |  | Reference |
| UAS-ATG8-mRFP | II | E. Hafen | [[6](#_ENREF_6)] |
| UAS-CD8-GFP | III | BL 5137 | [[7](#_ENREF_7)] |
| UAS-LAMP-GFP | II | H. Krämer | [[8](#_ENREF_8)] |
| UAS-mito-GFP | II | BL 8442 | [[9](#_ENREF_9)] |
| Exon-Trap Line | Chromosome |  | Reference |
| dlg-GFP | X | Flytrap CC01936 | [[10](#_ENREF_10)] |

1. Saxton WM, Hicks J, Goldstein LS, Raff EC (1991) Kinesin heavy chain is essential for viability and neuromuscular functions in Drosophila, but mutants show no defects in mitosis. Cell 64: 1093-1102.

2. Spradling AC, Stern D, Beaton A, Rhem EJ, Laverty T, et al. (1999) The Berkeley Drosophila Genome Project gene disruption project: Single P-element insertions mutating 25% of vital Drosophila genes. Genetics 153: 135-177.

3. Gustafson K, Boulianne GL (1996) Distinct expression patterns detected within individual tissues by the GAL4 enhancer trap technique. Genome 39: 174-182.

4. Lin DM, Goodman CS (1994) Ectopic and increased expression of Fasciclin II alters motoneuron growth cone guidance. Neuron 13: 507-523.

5. McGuire SE, Mao Z, Davis RL (2004) Spatiotemporal gene expression targeting with the TARGET and gene-switch systems in Drosophila. Sci STKE 2004: pl6.

6. Köhler K, Brunner E, Guan XL, Boucke K, Greber UF, et al. (2009) A combined proteomic and genetic analysis identifies a role for the lipid desaturase Desat1 in starvation-induced autophagy in Drosophila. Autophagy 5: 980-990.

7. Lee T, Luo L (1999) Mosaic analysis with a repressible cell marker for studies of gene function in neuronal morphogenesis. Neuron 22: 451-461.

8. Pulipparacharuvil S, Akbar MA, Ray S, Sevrioukov EA, Haberman AS, et al. (2005) Drosophila Vps16A is required for trafficking to lysosomes and biogenesis of pigment granules. Journal of Cell Science 118: 3663-3673.

9. Horiuchi D, Barkus RV, Pilling AD, Gassman A, Saxton WM (2005) APLIP1, a kinesin binding JIP-1/JNK scaffold protein, influences the axonal transport of both vesicles and mitochondria in Drosophila. Curr Biol 15: 2137-2141.

10. Buszczak M, Paterno S, Lighthouse D, Bachman J, Planck J, et al. (2007) The carnegie protein trap library: a versatile tool for Drosophila developmental studies. Genetics 175: 1505-1531.
