# Supplementary material for: Spastic Paraplegia Mutation N256S in the Neuronal Microtubule Motor KIF5A Disrupts Axonal Transport in a Drosophila HSP Model
Source: PLoS Genet. 2012 Nov 29;8(11):e1003066. doi: 10.1371/journal.pgen.1003066 (PMC3510046; doi:10.1371/journal.pgen.1003066)
Supplement: Table S2 — Primary antibodies used for immunochemistry and western blot in this study. (DOCX) [file pgen.1003066.s003.docx]

**Table S2. Primary antibodies used for immunochemistry and western blot in this study**

| Antigen | Animal | Dilution | Donor | Reference |
| --- | --- | --- | --- | --- |
| Bruchpilot (Nc82) | mouse | 1:100 | DSHB* | [[1](#_ENREF_1)] |
| Cleaved Caspase-3 (Asp175) | rabbit | 1:2500 | NEB #9661 | New England Biolabs |
| CSP (DSCP-2) | mouse | 1:50 | DSHB | [[2](#_ENREF_2)] |
| DVGlut | rabbit | 1:1000 | H. Aberle | [[3](#_ENREF_3)] |
| Even-skipped (3C10) | mouse | 1:10 | DSHB | [[4](#_ENREF_4)] |
| Khc (SUK4) | mouse | 1:100 | DSHB | [[5](#_ENREF_5)] |
| Synapsin (3C11) | mouse | 1:5 | DSHB | [[6](#_ENREF_6)] |
| β-Tubulin (E7) | mouse | 1:500 | DSHB | [[7](#_ENREF_7)] |

*DSHB Developmental Studies Hybridoma Bank

1. Wagh DA, Rasse TM, Asan E, Hofbauer A, Schwenkert I, et al. (2006) Bruchpilot, a protein with homology to ELKS/CAST, is required for structural integrity and function of synaptic active zones in Drosophila. Neuron 49: 833-844.

2. Zinsmaier KE, Eberle KK, Buchner E, Walter N, Benzer S (1994) Paralysis and early death in cysteine string protein mutants of Drosophila. Science 263: 977-980.

3. Mahr A, Aberle H (2006) The expression pattern of the Drosophila vesicular glutamate transporter: A marker protein for motoneurons and glutamatergic centers in the brain. Gene Expression Patterns 6: 299-309.

4. Wilson MJ, Dearden PK (2009) Tailless patterning functions are conserved in the honeybee even in the absence of Torso signaling. Dev Biol 335: 276-287.

5. Ingold AL, Cohn SA, Scholey JM (1988) Inhibition of kinesin-driven microtubule motility by monoclonal antibodies to kinesin heavy chains. J Cell Biol 107: 2657-2667.

6. Klagges BR, Heimbeck G, Godenschwege TA, Hofbauer A, Pflugfelder GO, et al. (1996) Invertebrate synapsins: a single gene codes for several isoforms in Drosophila. J Neurosci 16: 3154-3165.

7. Klymkowsky MW, Maynell LA, Polson AG (1987) Polar asymmetry in the organization of the cortical cytokeratin system of Xenopus laevis oocytes and embryos. Development 100: 543-557.
