# Supplementary material for: Spastic Paraplegia Mutation N256S in the Neuronal Microtubule Motor KIF5A Disrupts Axonal Transport in a Drosophila HSP Model
Source: PLoS Genet. 2012 Nov 29;8(11):e1003066. doi: 10.1371/journal.pgen.1003066 (PMC3510046; doi:10.1371/journal.pgen.1003066)
Supplement: Table S3 — Secondary antibodies used for immunochemistry and western blot in this study. (DOCX) [file pgen.1003066.s004.docx]

**Table S3. Secondary antibodies used for immunochemistry and western blot in this study**

| Antigen | Animal | Dilution | Source |
| --- | --- | --- | --- |
| HRP-Cy3 | goat | 1:500 | Dianova |
| HRP-Cy5 | goat | 1:500 | Dianova |
| mouse-Alexa-488 | goat | 1:500 | Molecular Probes |
| rabbit-Alexa-488 | goat | 1:500 | Molecular Probes |
| mouse-Alexa-568 | goat | 1:500 | Molecular Probes |
| rabbit-Alexa-568 | goat | 1:500 | Molecular Probes |
| rabbit-Atto 647 | goat | 1:500 | Molecular Probes |
| mouse-HRP-conjugated | goat | 1:2000 | Santa Cruz Biotechnology |
| rabbit-HRP-conjugated | goat | 1:2000 | Santa Cruz Biotechnology |
