# Supplementary material for: Spastic Paraplegia Mutation N256S in the Neuronal Microtubule Motor KIF5A Disrupts Axonal Transport in a Drosophila HSP Model
Source: PLoS Genet. 2012 Nov 29;8(11):e1003066. doi: 10.1371/journal.pgen.1003066 (PMC3510046; doi:10.1371/journal.pgen.1003066)
Supplement: Text S1 — Supporting image quantification methods. (DOCX) [file pgen.1003066.s005.docx]

**Text S1**

**Supporting image quantification methods**

**Generation of kymographs**

Kymographs were extracted from representative movies for each genotype using ImageJ. In case residual movement of the larvae was observed, movies were aligned with the StackReg plugin. Next, the Multiple Kymograph plugin was used to build kymographs.

**Quantification of degeneration**

To score degeneration the subsynaptic reticulum (SSR), neuronal membranes (anti-HRP) and synaptic vesicles (SVs) were visualized. An evaluator, blinded to the genotypes utilized an additive scoring system to account for inhomogeneity of the neuronal membrane staining, the abundance of synaptic vesicles and the presence of synaptic footprints as signs of degeneration [[1](#_ENREF_1)]. For details on the scoring system see Fig. S1A-E. A healthy NMJ was assigned a total score of 0 (Fig. S1A). Slight inhomogeneity in SV staining resulted in a total score of 0.25 (Fig. S1B, arrowhead). The scoring system is additive. For example, inhomogeneous HRP staining at more than 30 % of the NMJ (Score: 0.25) and a generally weak staining of SV accompanied by a loss of SV from more than 20 % of the NMJ (Score: 0.75) resulted in an overall score of 1 (Fig. S1C, arrowheads). NMJs with severe inhomogeneity of HRP staining in addition to almost complete loss of SV and presence of a least one synaptic footprint (Fig. S1D arrow) scored 3 (Fig. S1E).

To visualize the subsynaptic reticulum a GFP insertion in the discs-large locus [[2](#_ENREF_2)] that results in the expression of Dlg-GFP under the control of its endogenous promoter (Flytrap: CC01936) was used. Neuronal membranes in *Drosophila* were visualized with an antibody against horseradish peroxidase (HRP). SV vesicles were stained using a m-α-Synapsin antibody.

**Analysis of motoneuron cell loss**

To mark motoneuron cell bodies staining for the transcription factor Even-skipped (eve) was performed. Even-skipped is expressed in the medially located aCC, RP2 motor neurons (Fig. S1F) as well as in the lateral U/CQs motoneurons. It is also expressed in other neuronal cells, such as the medially located pCC interneurons and the EL interneuron cluster [[3](#_ENREF_3)]. To score motoneuron cell loss we quantified the aCC and RP2 motoneurons located in the ventral nerve cord middle line that innervates segments A1-A3 (anterior segments) and A5-A7 (posterior segments).

1. Eaton BA, Fetter RD, Davis GW (2002) Dynactin is necessary for synapse stabilization. Neuron 34: 729-741.

2. Buszczak M, Paterno S, Lighthouse D, Bachman J, Planck J, et al. (2007) The carnegie protein trap library: a versatile tool for Drosophila developmental studies. Genetics 175: 1505-1531.

3. Landgraf M, Jeffrey V, Fujioka M, Jaynes JB, Bate M (2003) Embryonic origins of a motor system: motor dendrites form a myotopic map in Drosophila. PLoS Biol 1: E41.
